# Transcriptional analysis of sweet orange trees co-infected with ‘*Candidatus* Liberibacter asiaticus’ and mild or severe strains of *Citrus tristeza virus*

**DOI:** 10.1186/s12864-017-4174-8

**Published:** 2017-10-31

**Authors:** Shimin Fu, Jonathan Shao, Cristina Paul, Changyong Zhou, John S. Hartung

**Affiliations:** 1 Citrus Research Institute, Southwest University, Chongqing, China; 2 United States Department of Agriculture-Agricultural Research Service, Molecular Plant Pathology Laboratory, Beltsville, MD USA

**Keywords:** Huanglongbing, CTV, HLB, Tristeza, Transcriptome, Host-pathogen interaction

## Abstract

**Background:**

Citrus worldwide is threatened by huanglongbing (HLB) and tristeza diseases caused by ‘*Candidatus* Liberibacter asiaticus’ (CaLas) and *Citrus tristeza virus* (CTV). Although the pathogens are members of the *α-proteobacteria* and *Closteroviridae*, respectively, both are restricted to phloem cells in infected citrus and are transmitted by insect vectors. The response of sweet orange to single infection by either of these two pathogens has been characterized previously by global gene expression analysis. But because of the ubiquity of these pathogens where the diseases occur, co-infection by both pathogens is very common and could lead to increased disease severity based on synergism. We therefore co-inoculated sweet orange trees with CaLas and either a mild or a severe strain of CTV*,* and measured changes of gene expression in host plants.

**Results:**

In plants infected with CaLas-B232, the overall alteration in gene expression was much greater in plants co-inoculated with the severe strain of CTV, B6, than when co-infected with the mild strain of CTV, B2. Plants co-infected with CaLas-B232 and either strain of CTV died but trees co-infected with CTV-B2 survived much longer than those co-infected with CTV-B6. Many important pathways were perturbed by both CTV-B2/CaLas-B232 and/or CTV-B6/CaLas-B232, but always more severely by CTV-B6/CaLas-B232. Genes related to cell wall modification and metal transport responded differently to infection by the pathogens in combination than by the same pathogens singly. The expressions of genes encoding phloem proteins and sucrose loading proteins were also differentially altered in response to CTV-B2 or CTV-B6 in combination with CaLas-B232, leading to different phloem environments in plants co-infected by CaLas and mild or severe CTV.

**Conclusions:**

Many host genes were expressed differently in response to dual infection as compared to single infections with the same pathogens. Interactions of the pathogens within the host may lead to a better or worse result for the host plant. CTV-B6 may exert a synergistic effect with CaLas-B232 in weakening the plant; on the other hand, the responses activated by the mild strain CTV-B2 may provide some beneficial effects against *Ca*Las-B232 by increasing the defense response of the host.

**Electronic supplementary material:**

The online version of this article (10.1186/s12864-017-4174-8) contains supplementary material, which is available to authorized users.

## Background

Huanglongbing (HLB) and Tristeza are destructive and globally distributed citrus diseases, and are responsible for tremendous economic losses to citrus industries worldwide [8, 60]. HLB is strongly associated with ‘Ca. Liberibacter asiaticus’ (CaLas), a gram-negative and phloem-limited member of the *α-Proteobacteria*. CaLas has a global distribution and can be transmitted through grafting and by the citrus psyllid, *Diaphorina citri* [8]. Tristeza is caused by *Citrus tristeza virus* (CTV), a member of the *Closteroviridae* with a single-stranded and positive sense RNA-genome of 19.3 kb [16]. Like CaLas, CTV replicates in the phloem cells and may also be transmitted by grafting and by several aphids, most notably *Toxoptera citricidus* [60].

HLB has been studied for more than 100 years. Early observations of what is likely to have been HLB were made in the late 1880s by growers in Chaoshan, China and in the Pearl River Delta area of Guangdong in China. The earliest unambiguous report of HLB and association of the disease with the citrus psyllid was however made from India. Since CaLas has not been cultured in vitro, Koch’s postulates have not been completed. The first *Ca*Las genomic sequence (Psy62) was obtained through multiple displacement amplification [22]. Genomes of additional six CaLas strains and three CaLam strains have been successively sequenced and are available at NCBI.

Considerable research has focused on host responses to infection. Transcriptome studies of host responses to HLB were conducted in leaves [24], fruits [47, 56], stems and roots [5, 92], as well as in resistant and susceptible varieties [3, 10, 26, 54]. Differentially expressed genes (DEGs) and altered metabolic pathways were described. Pathways related to sugar and starch metabolism, cell wall metabolism, secondary metabolism, hormone signaling, photosynthesis, phloem transport and ion uptake were found to be affected in citrus plants infected by CaLas.

Strains of CTV vary from very mild, which do not cause notable damage to citrus hosts, to severe strains that induce symptom patterns that include leaf yellowing, stiffening, and cupping. These symptoms are very similar to those associated with alterations in sucrose metabolism in the phloem and decline of roots [35, 41] and collapse of phloem tissue [29, 60] seen in HLB. Previously we have analyzed the transcriptome of sweet orange, *Citrus sinensis,* singly inoculated with CaLas-B232, mild CTV-B2 or severe CTV-B6 [30]. CTV-B2 does not induce visible symptoms in sweet orange while both CTV-B6 and CaLas-B232 are lethal. We found that the circadian rhythm system of *C. sinensis* and ionic balances were perturbed by all three pathogens but to differing degrees. Defense responses related to cell wall modification, regulation of transcription, hormone metabolism, secondary metabolism, kinases and both biotic and abiotic stress were activated by all three pathogens but with different patterns [30]. These results provided some valuable insight into the similarity and differences of the responses of *C. sinensis* to CaLas and CTV. Both CTV and CaLas are globally distributed, and are present simultaneously in most locations. Therefore, co-infection by strains of CTV and CaLas is now a common situation. In the current study, transcriptome profiles of *C. sinensis* co-infected with CTV-B2/CaLas-B232 (B2/B232) and CTV-B6/CaLas-B232 (B6/B232) were analyzed to identify differentially expressed gene (DEGs) and unveil how mild CTV-B2 and severe CTV-B6 affect the genome-wide gene expression changes of *C. sinensis* infected with CaLas-B232.

## Results

**Fig. 1:**
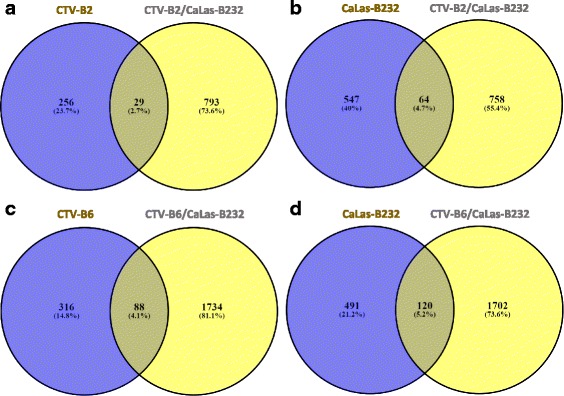
Differentially expressed transcripts in sweet orange responding to single and co-infection by CTV-B2, CTV-B6 and CaLas-B232. **a** venn diagram of differentially expressed transcripts (DEGs) in sweet orange responding to CTV-B2 and CTV-B2/CaLas-B232; **b** venn diagram of DEGs in sweet orange responding to CaLas-B232 and CTV-B2/CaLas-B232; **c** venn diagram of DEGs in sweet orange responding to CTV-B6 and CTV-B6/CaLas-B232; **d** venn diagram of DEGs in sweet orange responding to CaLas-B232 and CTV-B26CaLas-B232

40–45 million reads/sample were obtained and mapped to the *C. sinensis* reference genome [85], and about 60% of reads mapped successfully to the healthy, CTV-B2/CaLas-B232 and CTV-B6/CaLas-B232 sweet orange transcriptomes (Additional file 1: Figure S1). Differentially expressed transcripts (DETs) for CTV-B2/CaLas-B232 and CTV-B6/CaLas-B232 were identified by comparison with the transcriptome of the healthy controls. The number of up- or down regulated DETs varied for each co-infection (Additional file 2: Figure S2), and the overall alteration of the transcriptome was much greater in the presence of CTV-B6 than CTV-B2. We observed 402 and 420 transcripts induced and repressed respectively in response to CTV-B2/CaLas-B232, while 813 and 1009 transcripts were induced and repressed, respectively in response to CTV-B6/CaLas-B232. We compared the expression of the sweet orange genome in response to single infection and in combination after co-infection. We found that 29 transcripts were differentially regulated in response to CTV-B2 alone and in combination with CaLas-B232, while about twice that number were differentially regulated in response to CaLas-B232 alone and in combination with CTV-B2 (Fig. 1a and b). The number of transcripts that were co-regulated in response to CaLas-B232 alone and in combination with CTV-B6 was greater than the number that were co-regulated in response to CTV-B6 alone and in combination with CaLas-B232 (Fig. 1c and d).

**Fig. 2:**
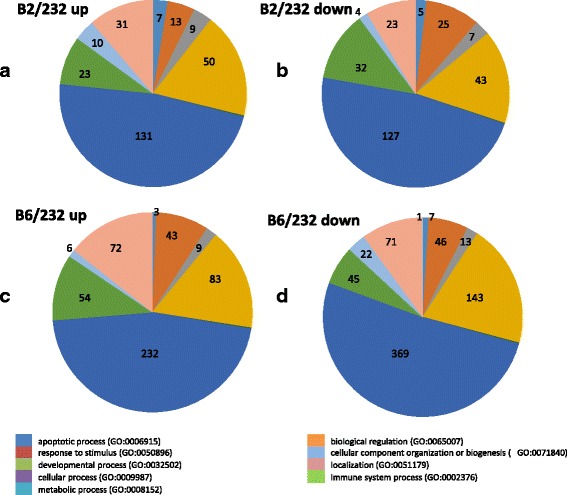
Categorization of differentially expressed transcripts in *Citrus sinensis* infected with CTV-B2/CaLas-B232 or CTV-B6/CaLas-B232 based on gene ontology. **a** and **c**, Induced transcripts; **b** and **d**, Repressed transcripts; B2/232, CTV-B2/CaLas-B232; B6/232, CTV-B6/CaLas-B232

**Fig. 3:**
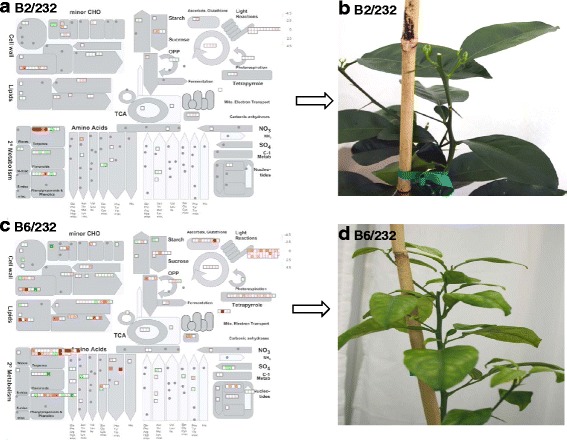
Metabolic overview in young leaves and subsequent symptoms in response to CTV-B2/CaLas-B232 and CTV-B6/CaLas-B232. **a** and **b**, CTV-B2/CaLas-B232; **c** and **d**, CTV-B6/CaLas-B232

**Fig. 4:**
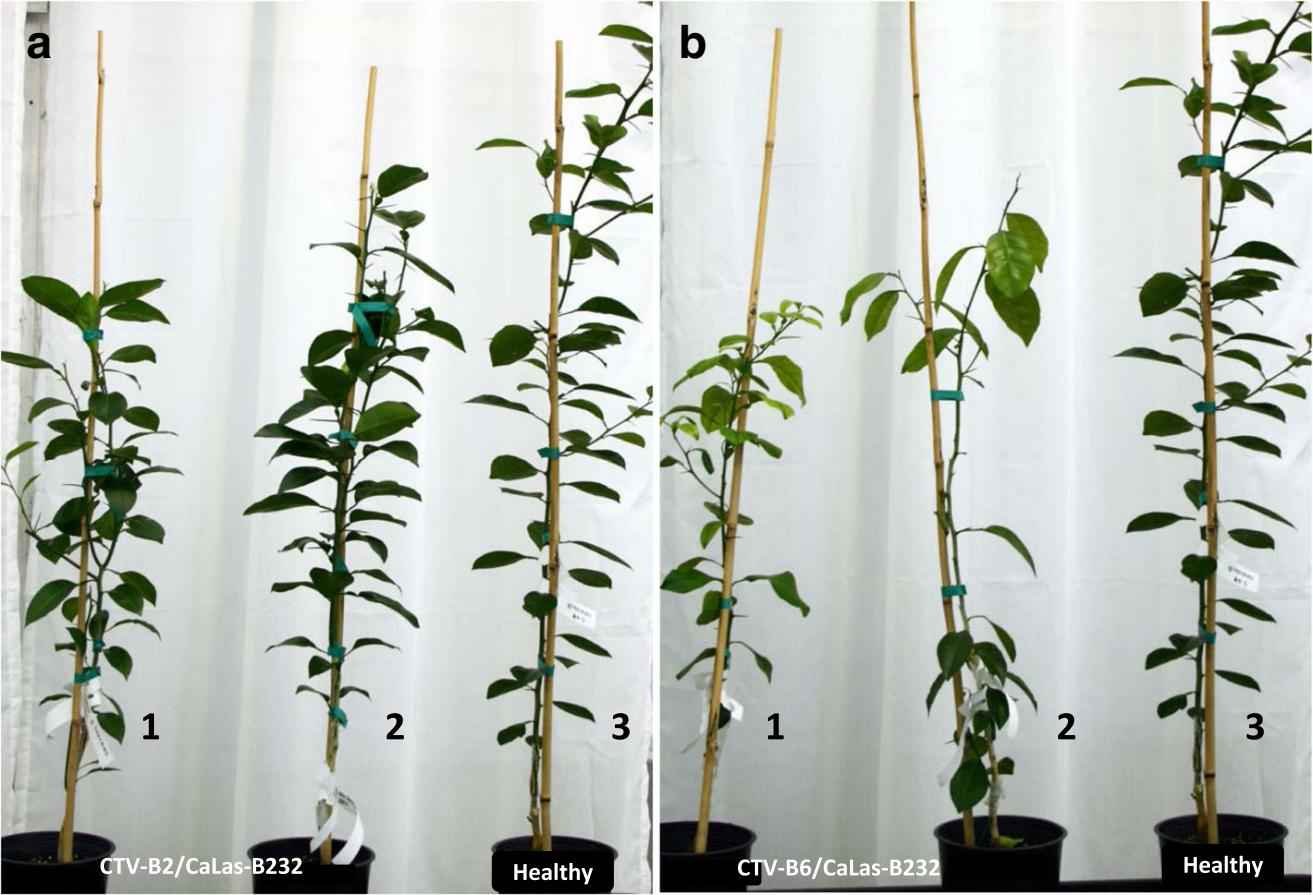
General appearance of sweet orange co-infected with CTV-B2/CaLas-B232 or CTV-B6/CaLas-B232. Pictures were taken 16 months after inoculation with CaLas and 13 months after inoculation with CTV. **a**, Trees co-infected with CTV-B2 and CaLas-B232; **b**, Trees co-infected with CTV-B6 and CaLas-B232; 1 and 2, Most and least severe symptom expression for each treatment; 3, Self-inoculated healthy control

The DETs were functionally placed in different Gene Ontology (GO) biological process (BP) categories. The largest numbers of genes induced by both CTV-B2/CaLas-B232 and/or CTV-B6/CaLas-B232 were associated with metabolic processes, followed by cellular processes, localization, biological regulation and responses to stimulus. Genes repressed by CTV-B2/CaLas-B232 and CTV-B6/CaLas-B232 also had a similar distribution within GO categories (Fig. 2). Many important metabolic pathways were heavily perturbed by CTV-B2/CaLas-B232, especially by CTV-B6/CaLas-B232 (Fig. 3a and c) in the young leaves that were sampled prior to the development of symptoms. These included metabolism of carbon compounds such as starch/sucrose and amino acids. Pathways related to photosynthesis, plant-pathogen interactions, hormone metabolism, signal transduction, circadian rhythm and ribosomal structure were also perturbed (Fig. 3a, c and Additional file 3: Tables S1 and Additional file 4: Table S2). The changes in gene expression are consistent with the symptoms that developed in mature leaves (Fig. 3b and d). Sixteen months after the inoculation of CaLas and 13 months the inoculation of CTV, the trees with the most and least severe symptoms (Fig. 4) were selected and compared to the self-inoculated healthy controls. Trees confirmed to be infected with both pathogens, either CTV-B2/CaLas-B232 or CTV-B6/CaLas-B232, were all smaller than the healthy controls. Trees infected with CTV-B6/CaLas-B232 (Fig. 4b) were also much weaker and showed much more severe symptoms, including chlorosis, stunting and leaf curling than plants infected with CTV-B2/CaLas-B232 (Fig. 4a).

### Validation of RNA sequence data by RT-qPCR

**Fig. 5:**
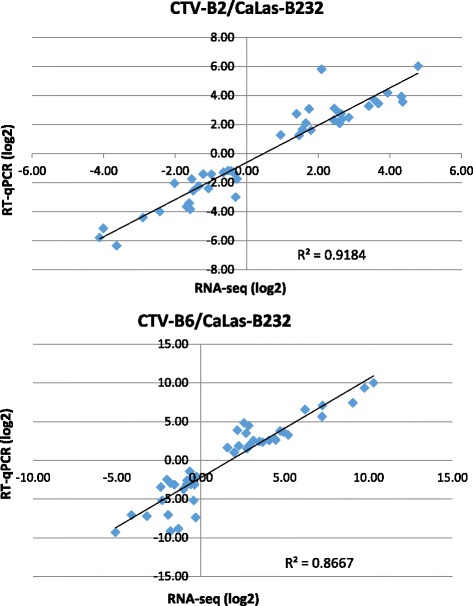
Correlation of estimates of differential gene expression by RT-qPCR and RNA-seq. **FC**: Log_2_ Fold Change

Gene expression analyses were performed with RT-qPCR to verify RNA sequence data. In total, 60 genes involved in different biological processes were selected, including genes encoding transporters ZIP1, ZIP5, ZIP11, COPT1, PHT2;1 and OPT7, transcription factors (TF) WRKY33, WRKY46, WRKY70 and MYB77, hormone metabolism proteins LOX3, GA2OX8, TPS21, phloem protein PP2-B15, light reaction proteins LHCA4 and LHCB6, cytochrome P450 members CYP83B1 and CYP94B1, light signaling related ELIP1, lipid metabolism protein LP1, an F-box family protein, and CCT-motif family proteins related to regulation of transcription PCL1 and CBF4. Primer information is provided in Additional file 5: Table S3. Similar expression patterns were revealed by both techniques for these genes. Spearman’s rho values of 0.91 for CTV-B2/CaLas-B232 and 0.87 for CTV-B6/ CaLas-B232 (Fig. 5) and low variation within the replicated samples (Additional file 6: Figure S3) confirmed the reliability of RNA sequence data.

### Photosynthesis, carbohydrate and amino acid metabolism

**Fig. 6:**
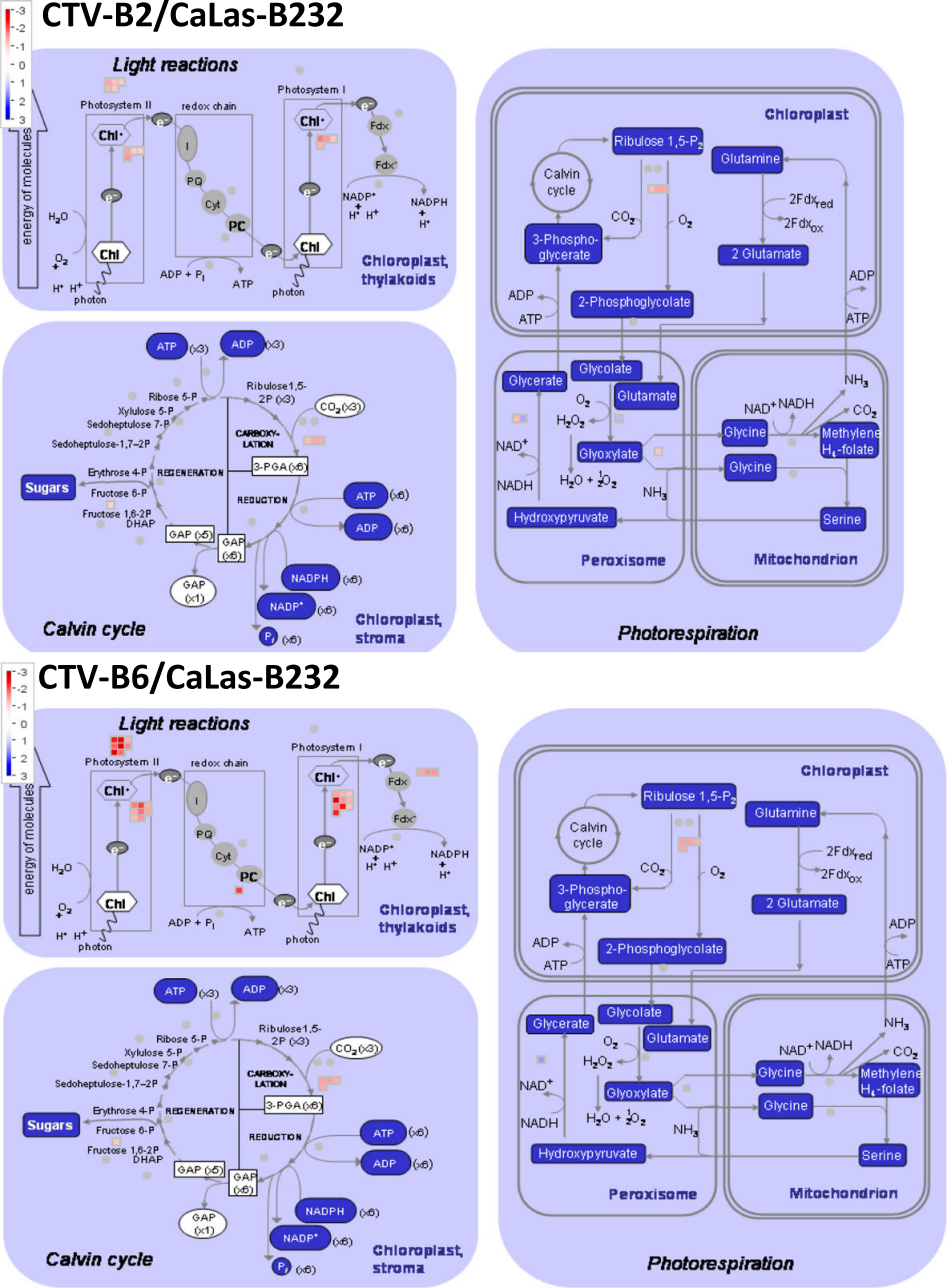
Photosynthetic system of *Citrus sinensis* perturbed by co-infection with CTV-B2/CaLas-B232 and CTV-B6/CaLas-B232

We found that many DETs associated with the light reactions of photosynthesis, Calvin cycle and photorespiration were repressed by CTV-B2/CaLas-B232 and/or CTV-B6/CaLas-B232 (Fig. 6). In photosystem II these genes included chlorophyll binding proteins (LHB1B1, LHCA1 and LHCA2), light harvesting complex (LHCB3, LHCB5, LHCB6 and LHCA4) and calcium ion binding proteins (PPL1 and PSBQ). In photosystem I subunits (PSAD-1, PSAF, PSAO, PSAD-2, PSAH-1, PSAL, PSAK, PSAP and PSAO), rubisco activase (RCA) and plastid transcriptionally active14 (PTAC14) were repressed (Additional file 3: Table S1 and Additional file 4: Table S2). The repression of genes involved with the synthesis of tetrapyrrole (Additional file 7: Figure S4) and the light harvesting complex of the chloroplast is consistent with reduced capacity for photosynthesis, and is consistent with the symptoms that subsequently developed in the plants (Fig. 3). Symptoms observed following co-infection by CTV-B6 and CaLasB232 were similar to those produced by either pathogen alone (Additional file 8: Figure S5).

Metabolism of amino acids including aspartate, proline, arginine, methionine, cysteine, asparagine, tyrosine and tryptophan was also disrupted by CTV-B2/CaLas-B232 and CTV-B6/CaLas-B232. Both nitrogen (N) and sulfur (S) assimilation are linked with amino acid metabolism. For example, *SERAT1;1* (serine acetyltransferase1;1) and *SERAT2;2* involved in cysteine synthesis were induced by both CTV-B2/CaLas-B232 and CTV-B6/CaLas-B232 (Additional file 3: Table S1 and Additional file 4: Table S2). Glutamate synthase (NADH) (GLT1), involved in N-metabolism, was repressed by both CTV-B2/CaLas-B232 and CTV-B6/CaLas-B232. Intriguingly, several genes associated with S-assimilation were exclusively activated by CTV-B6/CaLas-B232 rather than CTV-B2/CaLas-B232. These included APS1 (ATP sulfurylase 1), APS2 and APR1 (APS reductase 1). AKN1 (APS kinase) was repressed by CTV-B6/CaLas-B232 but was not affected by CTV-B2/CaLas-B232 (Additional file 3: Table S1 and Additional file 4: Table S2).

### Cell wall

The primary cell wall of a land plant is composed of the major carbohydrates cellulose, hemicelluloses and pectin. Genes encoding pectin methyl esterase were generally repressed, whereas genes related to cellulose synthesis were induced by CTV-B6/CaLas-B232.

### Transport and heavy metal ions

The transportation system of the host plants was heavily disturbed by CTV-B6/CaLas-B232 and CTV-B2/CaLas-B232. Genes encoding sugar transporters were up-regulated in young leaves in response to co-infection. Transportation of metals was impaired by CTV-B2/CaLas-B232 and/or CTV-B6/CaLas-B232 as inferred from the suppression of iron, copper and zinc transporter genes (Additional file 3: Table S1 and Additional file 4: Table S2). The expression of phloem protein PP2-B15 was highly up-regulated in response to CTV-B6/CaLas-B232 (Additional file 4: Table S2), but no alteration was observed in response to CTV-B2/CaLas-B232.

### The circadian system

**Fig. 7:**
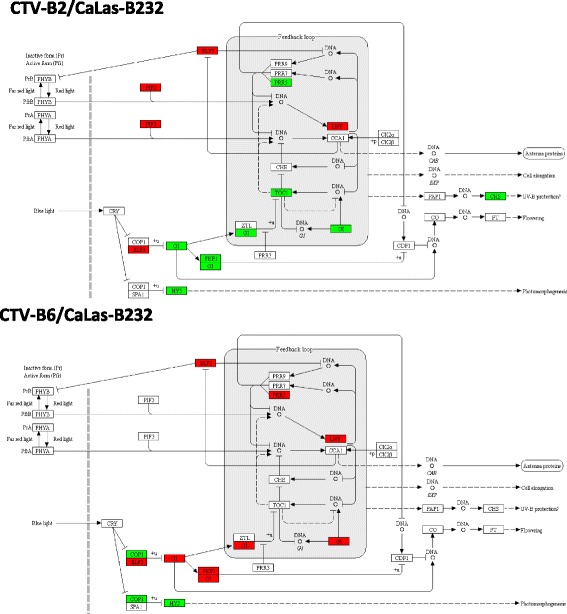
Disturbances in the circadian system by CTV-B2/CaLas-B232 and CTV-B6/CaLas-B232. Red boxes, down- regulated genes; Green boxes, up-regulated genes

The plant circadian clock regulates expression of as much as one-third of the *Arabidopsis* genome [71], including carbohydrate metabolism and defense responses [33, 37, 69]. The clock is composed of interlocking transcription-translation feedback loops. These were greatly disrupted by CTV-B2/CaLas-B232 and CTV-B6/CaLas-B232 (Fig. 7).

### Hormones and control of defense responses

The metabolism of abscisic acid, auxins, brassinosteroids, cytokinins, ethylene, gibberellins, jasmonate and salicylic acid were all perturbed by CTV-B6/CaLas-B232 and to a lesser extent by CTV-B2/CaLas-B232 (Table 1; Additional file 9: Figure S6). A large number of transcription factors (TF) were affected by the pathogens, including AP2/EREBP, basic helix-loop-helix (bHLH), C2C2 (Zn), MYB, WRKY, bZIP, PHOR1 and Pseudo ARR (PRR) (Additional file 3: Tables S1 and Additional file 4: Table S2). An overwhelming majority of genes regulated by AP2/EREBP (APETALA/ETHYLENE RESPONSIVE ELEMENT BINDING PROTEIN) were down-regulated in response to CTV-B2/CaLas-B232 and CTV-B6/CaLas-B232. These included ethylene-responsive element binding factor (ERF13 and ERF38) and the C-repeat-binding factors (CBF4, CBF1 and CBF2). MYB14, MYB73 and MYB77 were all repressed in response to both CTV-B2/CaLas-B232 and CTV-B6/CaLas-B232. Ethylene signaling and ethylene mediated defense responses were activated in response to CTV-B2/CaLas-B232 and CTV-B6/CaLas-B232 (Additional file 3: Table S1 and Additional file 4: Table S2), as well as to CTV-B2, CTV-B6 and CaLas-B232 separately.

**Table 1:** Responses of hormones to infection by CTV-B2, CTV-B6 and CaLas-B232

Categories	CTV-B2^a^	CTV-B6^a^	CaLas-B232^a^	CTV-B2/ CaLas-B232^b^	CTV-B6/ CaLas-B232^b^
Abscisic acid (ABA)		Y	Y	Y	Y
Auxin			Y	Y	Y
Brassinosteroid				Y	Y
Cytokinin (CK)					Y
Ethylene	Y	Y	Y	Y	Y
Gibberelin (GA)		Y		Y	Y
Jasmonate (JA)			Y	Y	Y
Salicylic acid (SA)	Y	Y	Y		Y

a: sweet orange trees were singly inoculated with pathogens (Fu et al., 2016)

b: sweet orange trees were doubly inoculated with CaLas-B232 and mild CTV-B2 or severe CTV-B6

Y: hormone related pathways were disrupted by single or dual pathogen infection

### Alteration of ribosomal composition

Ribosomal proteins play crucial roles in cell division, growth and metabolism in plants. Several members of ribosomal protein families were down-regulated in host plants in response to dual infection by CTV-B2/CaLas-B232 but many more by CTV-B6/CaLas-B232 (Table 2).

**Table 2:** Transcripts encoding ribosomal proteins were differentially regulated in *Citrus sinensis* in response to infection by CTV-B2/CaLas-B232 and CTV-B6/CaLas-B232

Gene symbol	Transcript id_	AGI	Gene description	Log 2 Fold-change	
	*Citrus sinensis*			CTV-B2/ CaLas-B232	CTV-B6/ CaLas-B232
RPS5	orange1.1g021869m	AT2G33800	ribosomal protein S5 family protein		−1.42
RPS5	orange1.1g013474m	AT1G64880	ribosomal protein S5 family protein		−1.03
RPS6	orange1.1g027743m	AT1G64510	ribosomal protein S6 family protein		−1.71
RPS9	orange1.1g042358m	AT1G74970	ribosomal protein S9		−1.46
RPS10	orange1.1g041275m	AT3G13120	30S ribosomal protein S10, chloroplast, putative		−1.70
RPS13	orange1.1g030930m	AT5G14320	30S ribosomal protein S13, chloroplast (CS13)		−1.39
RPS17	orange1.1g033970m	AT1G79850	ribosomal protein S17		−1.48
RPS20	orange1.1g029900m	AT3G15190	chloroplast 30S ribosomal protein S20, putative	−1.11	−1.69
RPS1	orange1.1g015066m	AT5G30510	ribosomal protein S1		−1.57
GHS1/S21	orange1.1g030080m	AT3G27160	glucose hypersensitive1, structural constituent of ribosome		−1.86
RPS4B	orange1.1g024793m	AT5G07090	40S ribosomal protein S4 (RPS4B)		−1.12
RPS5A	orange1.1g028585m	AT3G11940	ribosomal protein 5A, structural constituent of ribosome		−1.16
RPS8A	orange1.1g044642m	AT5G20290	40S ribosomal protein S8 (RPS8A)		−1.06
RPS13A	orange1.1g031857m	AT4G00100	*Arabidopsis thaliana* ribosomal protein S13A		−1.02
RPS16A	orange1.1g032049m	AT2G09990	40S ribosomal protein S16		−1.04
RPS19C	orange1.1g032099m	AT5G61170	40S ribosomal protein S19 (RPS19C)		−1.04
RPS20B	orange1.1g033482m	AT3G47370	40S ribosomal protein S20 (RPS20B)		−1.14
RPS23B	orange1.1g032383m	AT5G02960	40S ribosomal protein S23 (RPS23B)		−1.46
RPS24B	orange1.1g032727m	AT5G28060	40S ribosomal protein S24 (RPS24B)		−1.77
RPS25	orange1.1g033931m	AT4G34555	40S ribosomal protein S25, putative		−1.48
RPS26C	orange1.1g032843m	AT3G56340	40S ribosomal protein S26 (RPS26C)		−1.13
PSRP3	orange1.1g030605m	AT1G68590	plastid-specific 30S ribosomal protein 3, putative / PSRP-3		−1.33
PSRP3	orange1.1g029878m	AT5G15760	plastid-specific 30S ribosomal protein 3, putative / PSRP-3		−1.44
PSRP3	orange1.1g030716m	AT1G68590	plastid-specific 30S ribosomal protein 3, putative / PSRP-3		−1.40
RPS30A	orange1.1g020955m	AT5G24490	30S ribosomal protein, putative	−1.09	−1.39
RPL3	orange1.1g023905m	AT2G43030	ribosomal protein L3 family protein		−1.64
RPL5	orange1.1g024440m	AT4G01310	ribosomal protein L5 family protein		−1.69
RPL6	orange1.1g027306m	AT1G05190	embryo defective 2394		−1.58
RPL9	orange1.1g029153m	AT3G44890	ribosomal protein L9		−1.81
RPL10	orange1.1g027713m	AT5G13510	ribosomal protein L10 family protein		−1.34
RPL13	orange1.1g026067m	AT1G78630	embryo defective 1473		−1.37
RPL15	orange1.1g024515m	AT3G25920	structural constituent of ribosome		−1.39
RPL17	orange1.1g041707m	AT3G54210	ribosomal protein L17 family protein		−1.79
RPL18	orange1.1g030804m	AT1G48350	ribosomal protein L18 family protein	−1.08	−1.94
RPL19	orange1.1g026573m	AT4G17560	ribosomal protein L19 family protein		−1.53
RPL21	orange1.1g027519m	AT1G35680	50S ribosomal protein L21, chloroplast/CL21		−1.72
RPL24	orange1.1g029417m	AT5G54600	50S ribosomal protein L24, chloroplast (CL24)		−1.98
RPL27	orange1.1g029509m	AT5G40950	ribosomal protein large subunit 27		−1.01
RPL28	orange1.1g031725m	AT2G33450	50S ribosomal protein L28, chloroplast (CL28)		−1.42
RPL29	orange1.1g030809m	AT5G65220	ribosomal protein L29 family protein		−1.32
RPL34	orange1.1g031306m	AT1G29070	ribosomal protein L34 family protein	−1.01	−2.10
RPL35	orange1.1g031865m	AT2G24090	ribosomal protein L35 family protein		−1.29
RPL3A	orange1.1g038172m	AT1G43170	Arabidopsis ribosomal protein 1		−1.61
RPL6B	orange1.1g026764m	AT1G74060	60S ribosomal protein L6 (RPL6B)		−1.17
RPL7C	orange1.1g026093m	AT2G44120	60S ribosomal protein L7 (RPL7C)		−1.14
RPL15A	orange1.1g028730m	AT4G16720	60S ribosomal protein L15 (RPL15A)		−1.16
RPL17B	orange1.1g030568m	AT1G67430	60S ribosomal protein L17 (RPL17B)		−1.17
RPL18C	orange1.1g030771m	AT5G27850	60S ribosomal protein L18 (RPL18C)		−1.27
RPL24A	orange1.1g031225m	AT2G36620	ribosomal protein L24		−1.05
RPL32A	orange1.1g032796m	AT4G18100	60S ribosomal protein L32A		−1.02
RPL37C	orange1.1g034400m	AT3G16080	60S ribosomal protein L37 (RPL37C)		−1.22
RPL7A	orange1.1g025016m	AT2G47610	60S ribosomal protein L7A (RPL7aA)		−1.02
RPL7A	orange1.1g024981m	AT3G62870	60S ribosomal protein L7A (RPL7aB)		−1.04
RPLP1	orange1.1g033944m	AT5G24510	60s acidic ribosomal protein P1, putative	1.92	1.75
RPP3A	orange1.1g033353m	AT4G25890	60S acidic ribosomal protein P3 (RPP3A)		−1.14
RPL18A	orange1.1g035537m	AT1G29970	60S ribosomal protein L18A-1		−1.40
RPL37A	orange1.1g044880m	AT3G10950	60S ribosomal protein L37a		−1.04
RRL13A	orange1.1g028649m	AT5G48760	60S ribosomal protein L13A	1.12	
BBC1/L13	orange1.1g028624m	AT3G49010	Arabidopsis thaliana breast basic conserved 1		−1.92
^a^	orange1.1g010232m	AT1G71720	S1 RNA-binding domain-containing protein		−1.21
^a^	orange1.1g034489m	AT5G40080	60S ribosomal protein-related		1.46
^a^	orange1.1g043340m	AT2G39670	radical SAM domain-containing protein		−1.58
^a^	orange1.1g044572m	AT3G27180	unknown protein		−1.01

Note: ‘^a^’ represents transcripts without gene symbol

## Discussion

Differentially expressed genes and altered metabolic pathways of hosts provide a broad perspective of the host response to infection by CaLas [2, 54, 92] and by CTV [14, 49, 86]. We have compared changes in transcription in sweet orange plants in response to mild and severe strains of CTV and to CaLas-B232 when inoculated separately, which provided an overview of how susceptible hosts respond to CTV and CaLas [30]. Because CTV and CaLas are globally distributed, it is very common to have trees infected with both pathogens. Thus single pathogen studies, while essential, do not provide all of the needed data. In this study we have collected and analyzed transcriptome data from sweet orange plants co-infected with CTV-B2 and CaLas-B232 or CTV-B6 and CaLas-B232. In preliminary experiments we found that trees became PCR-positive for CTV much more quickly and more reliably than for CaLas (data not shown), so that simultaneous inoculations with the two pathogens was not effective. We therefore inoculated trees with CaLas-B232 first, and after the trees became positive for CaLas by qPCR (about 3 months after of inoculation) and we inoculated the trees with CTV-B2 or CTV-B6. In this way we observed the host response to infection by both pathogens compared with each pathogen separately [30]. The alteration of the transcriptome in response to co-infection by both pathogens was much larger than in response to infection by any single pathogen [30]. Some genes showed similar expression patterns in both circumstances but others showed different, and even opposite expression patterns (Additional file 3: Table S1 and Additional file 4: Table S2). Although all trees died eventually, trees co-infected with CTV-B2 and CaLas-B232 were slower to develop symptoms and survived longer than trees co-infected with CTV-B6 and CaLas-B232 (Fig. 4). Defense responses in trees may have been activated by CTV-B2 to provide limited protection against CaLas-B232, while CTV-B6 and CaLas-B232 acted synergistically to rapidly kill the trees.

### Photosynthesis carbohydrate and amino acid metabolism

The most abundant tetrapyrrole in plants is chlorophyll, the pigment that harvests light for photosynthesis. Genes related to tetrapyrrole synthesis were repressed (Additional file 7: Figure S4) in young leaves prior to symptom development in response to CTV-B2/CaLas-B232 and especially by CTV-B6/CaLas-B232. These genes included *CHLI2* (magnesium chelatase I2), *FC1* (ferrochelatase 1) and *HEME2* (uroporphyrinogen decarboxylase) (Additional file 3: Table S1 and Additional file 4: Table S2). These pathways were also disrupted by infection with CaLas alone [2, 14, 30, 55]. Expression of some photosynthesis-related genes was increased in response to CTV-B6 and CaLas-B232 alone but no changes were found in response to CTV-B2 [30]. This indicates that the reduced capacity for photosynthesis as the host responds to the two pathogens is primarily in response to CaLas.

The HLB syndrome also includes impaired carbohydrate metabolism [2, 54, 55, 91], and similar effects have been reported in sweet orange trees infected with *Spiroplasma citri*, another phloem-limited bacterial pathogen [9]. Sucrose is transported from source to sink organs and starch is the storage reserve in plants. Metabolic pathways related to energy generation from carbohydrates, including glycolysis, the oxidative pentose phosphate pathway (OPP) and the tricarboxylic acid cycle (TCA), were all repressed by both CTV-B2/CaLas-B232 and CTV-B6/CaLas-B232 (Additional file 3: Table S1 and Additional file 4: Table S2). The OPP is the main source of NADPH. Both the respiratory TCA pathway and glycolysis are essential to produce energy and precursors of amino acids and secondary metabolites. The repression of these processes may limit plant growth and successful defense response as plant defense is energy intensive [7].

Amino acids are also involved in plant-pathogen interactions [74, 89]. Amino acid transporters are involved in plant defense responses by alteration of amino acid concentrations in balancing N pools. Our data strongly indicated that not only the metabolism of amino acids but also their related transporters were increased in response to dual infection, particularly by CTV-B6/CaLas-B232 (Additional files 3: Table S1 and Additional file 4: Table S2). Amino acid permeases (AAPs) are important for phloem transport of amino acids. *AAP6, AAP7* and *ProT1* (proline transporter1) were induced by CTV-B2/CaLas-B232; AAP6 and CAT8 (cationic amino acid transporter 8) were induced while *AAP3* was highly repressed by CTV-B6/CaLas-B232. AAP3 transports the basic amino acids lysine and arginine [28]. *AAP6* was expressed primarily in sink tissues [68] but also in xylem parenchyma in *Arabidopsi*s and AAP6 was predicted to transfer amino acids from the xylem to the phloem [39, 63]. Some studies have found that the abundance of amino acids is correlated positively with the performance and behavior of phloem feeding insects: higher levels of amino acids increased aphid performance in potato [43, 66]. Both CaLas and CTV are transmitted by phloem feeding insects, and CaLas has to obtain nutrients such as amino acids and enzymes from the host due to its greatly reduced genome [22, 38]. The altered expression of transporter genes may result in a traffic jam of carbohydrates, amino acids and other substances in the phloem. The availability and abundance of nutrients and the composition of the amino acids in phloem would be correlated with the growth of CaLas and favor feeding by the insect vectors.

### Cell wall

*CSLB04* and *CSLE1* associated with cellulose synthesis in cell walls were up-regulated while COBL7 (COBRA-like 7), which is thought to be required for properly oriented cell expansion [72, 73] was down-regulated in response to CTV-B6/CaLas-B232. In our earlier study, *COBL7* was strongly up-regulated by CTV-B2 [30]. Genes related to cell wall degradation were generally up-regulated in response to both CTV-B2/CaLas-B232 and CTV-B6/CaLas-B232. Genes encoding cell wall proteins, such as HRGP (hydroxyproline-rich glycoprotein), RGP2 (reversibly glycosylated polypeptide 2) and LRR (leucine-rich repeat family protein) were up-regulated in response to CTV-B2/CaLas-B232 and/or CTV-B6/CaLas-B232 (Additional file 3: Table S1 and Additional file 4: Table S2). Genes related to cell wall modification were differentially regulated in response to CTV-B2/CaLas-B232 and CTV-B6/CaLas-B232. *XTR6* (xyloglucan endotransglycosylase) was down-regulated in response to both CTV-B2/CaLas-B232 and CTV-B6/CaLas-B232 but was up-regulated in response to CTV-B2, CTV-B6 and CaLas-B232 when inoculated singly [30]. Expansin genes, *EXP1*, *EXP3*, *EXP4*, *EXPB5*, *EXPL1*, etc., were also differentially regulated by CTV-B2/CaLas-B232 and/or CTV-B6/CaLas-B232. *EXLB3* was especially up-regulated by CTV-B6/CaLas-B232 (Additional file 3: Table S1 and Additional file 4: Table S2).

### Transport and heavy metal ions

Genes encoding sugar transporters were up-regulated in young leaves in response to co-infection, as well as to CTV-B6 and CaLas-B232 alone [30]. The phloem-located SUC/SUT1 (sucrose-proton symporter2) is responsible for loading sucrose into sieve tubes [21, 75] and was induced in response to CTV-B2/CaLas-B232. Potassium ions also play a crucial role in the transport of photo assimilates in the phloem [34]. Potassium channel SKOR (cyclic nucleotide binding) was up-regulated in response to CTV-B6/CaLas-B232 (Additional file 4: Table S2). In *Arabidopsis* the loss of the potassium channel AKT2/3 impacted the loading of sugar into the phloem via the regulation of the phloem osmotic potential and the activity of sucrose transporters [18]. mRNAs for ATPases, CNG channels, ABC and nitrate transporters have been identified in sieve element sap of *Ricinus* and barley [20, 21]. These were also differentially expressed in response to CTV-B2/CaLas-B232 and CTV-B6/CaLas-B232.

FER1 (Ferric iron binding), radical SAM domain-containing protein, PCS2 (phytochelatin synthase 2) were down-regulated in response to CTV-B2/CaLas-B232 and/or CTV-B6/CaLas-B232 (Additional file 3: Table S1 and Additional file 4: Table S2). The over-expression of ferric reduction oxidase genes *FRO6*, *FRO7* and *PCS2* was found in response to CaLas-B232 but not to CTV-B2 or to CTV-B6 in the previous study [30]. This is consistent with iron deficiency in young leaves. 80% of leaf iron is located in the chloroplasts [78]. A shortage of iron leads to reduction of iron-containing proteins and most markedly to decreased production of chlorophyll. The deficiency of chlorophyll results in yellowing symptoms on leaves as observed on leaves infected with CaLas or CTV-B6 and also lowers the capacity for photosynthesis directly [1].

FP6 (Farnesylated protein 6) was induced by CTV-B2/CaLas-B232. FP3 and FP6 also possess the heavy metal binding MXCXXC domain and are involved in Cu(II) and Zn(II) transport in *Arabidopsis* roots [31]. HMA5 (Heavy metal ATPase) also contains the MXCXXC domain and functions in copper (Cu(II)) detoxification [4]. The expression of these metal handling proteins is consistent with deficiency of metal ions, which may be exacerbated by the dramatic decline of fibrous roots after infection with CaLas [35, 41]. Symptoms of zinc-deficiency are often observed on new leaves following infection by CaLas [8, 76, 84].

Up-regulation of zinc and copper transporter genes was also found in response to CTV-B6 and CaLas-B232 when inoculated alone [30, 25, 54]. The expressions of ZIP1, ZIP2, ZIP4, ZIP5 and ZIP11 were all induced by CaLas-B232 and CTV-B6/CaLas-232, while only ZIP5 and ZIP11 were induced by CTV-B2/CaLas-B232. ZIP6 was also induced by CTV-B6/CaLas-B232 and the expression level of these ZIPs was much higher in response to CTV-B6/CaLas-B232 than to CTV-B2/CaLas-B232 or CaLas-B232 alone. Interestingly, the expression of ZIP1, ZIP4 and ZIP5 was repressed by CTV-B2. Both CaLas and the severe strain of CTV, B6, cause chlorosis and symptoms of zinc deficiency on leaves, phloem collapse, and decline of roots, but the mild strain of CTV-B2 does not cause any damage or symptoms on indicator plants.

P-proteins (Phloem-proteins) are components of phloem involved both in the long-distance movement of macromolecules and in signaling [44, 52]. Several phloem proteins PP2-B10, PP2-B11, PP2-B13 and PP2-B15 were over expressed in response to CTV-B6 or CaLas-B232 alone, but PP2-B13 and PP2-B15 were dramatically down-regulated by CTV-B2 [30]. However, no change in expression of PP2-B13 and PP2-B15 was found after co-infection with CTV-B2/CaLas-B232. PP2-B15 has been previously reported to be induced in hosts in response to CaLas [45, 47, 54]. The increased expression of PP2-B13 and PP2-B15 by CaLas appears to be offset by the interaction with CTV-B2. This is also solid evidence that Zn transporters and phloem proteins were connected in a co-expression network as suggested [67, 92]. These results may also support the notion that CTV-B6 acts synergistically with CaLas-B232 to disrupt phloem transport and further stimulate the expression of ZIPs and PP2. Patterns of gene expression activated by CTV-B2 may provide some beneficial effects on infection by CaLas-B232. In addition, induction of ZIP4, ZIP5 and PP2-B15 may serve as biomarkers for HLB disease.

### The circadian system

The plant circadian clock regulates expression of as much as one-third of the *Arabidopsis* genome [71], including carbohydrate metabolism and defense responses [33, 37, 69]. The clock is composed of interlocking transcription-translation feedback loops. These were greatly disrupted by CTV-B2/CaLas-B232 and CTV-B6/CaLas-B232 (Fig. 7), as we observed in our previous study of the pathogens in single infections [30]. Of particular interest is GIGANTEA (GI), a key regulatory protein that is necessary to maintain the appropriate period and amplitude of *cca1* and *lhy* gene expression in the outer feedback loop of the circadian system in *Arabidopsis* [64]. GI also functions in the sucrose-signaling network of *Arabidopsis* [15], which probably contributes to the perturbations of sucrose metabolism described above. GI is also involved in the regulatory pathway that determines wall in-growth to provide physical barriers to limit pathogen invasion [13, 23]. Deformation of phloem cell walls is observed in citrus in response to infection by CaLas [29] and stem pitting, caused by in-growths of the phloem cells is a primary symptom of CTV [16]. The expression of GI was increased in response to CTV-B2/CaLas-B232, as well as to CTV-B2, CTV-B6 and CaLas-B232 alone [30], but was repressed by CTV-B6/CaLas-B232 together. The repression of GI would limit the production of intracellular barriers and lead to a weakened defense against the invasion of CaLas-B232 and CTV-B6. Trees inoculated with CaLas-B232 and CTV-B6 together tended to die rapidly rather than linger in diseased states as when inoculated with CTV-B6 or CaLas-B232 alone. Other genes of the circadian system were also differentially regulated in response to CTV-B2/CaLas-B232 and/or CTV-B6/CaLas-B232. These included ELF3 (early flowering), PIF3 (phytochrome interacting factor), and COP1 (constitutive photo morphogenesis protein 1). ELF3 and COP1 control the stability of GI and therefore mediate photoperiodic flowering and circadian function [88]. Whether plants were infected with CaLas and CTV separately or together, the circadian system was heavily disturbed. Alterations of the circadian system may contribute to the generalized reprogramming of the transcriptome observed in response to these pathogens, and has been observed previously for CaLas but not for CTV [30, 47].

### Hormones and control of defense responses

The central plant immune responses associated with jasmonic acid, salicylic acid and ethylene [65] interact with plant hormones, including gibberellins [17], abscisic acid [57], auxins [40, 62, 83], cytokinins [40] and brassinosteroids 61]. Pathways leading to production of these hormones were also affected by CaLas-B232 alone [30]. The brassinosteroid, cytokinin and gibberellic acid, ethylene, abscisic acid and salicylic acid associated pathways were affected by CTV-B6 but only the ethylene and SA related pathways were affected by CTV-B2 (Table 1). Lipoxygenase 2 (LOX2) associated with JA synthesis-degradation was highly induced by CaLas-B232 alone but only slightly induced by CTV-B2/CaLas-B232 and repressed by CTV-B6/CaLas-B232 (Additional file 4: Table S2).

The WRKY factors are important regulators of SA-dependent defense responses [82] and some of them have been demonstrated in SA-JA crosstalk. Genes encoding WRKY transcription factors were mostly down-regulated in response to CTV-B2/CaLas-B232 and/or CTV-B6/CaLas-B232 (Additional file 3: Tables S1 and Additional file 4: Table S2), but WRKY 50, WRKY60 and WRKY70 were induced by CTV-B6/CaLas-B232, as well as by CTV-B2, CTV-B6 and CTV-B232 alone [30]. WRKY70, WRKY11 and WRKY17 are involved in SA/JA crosstalk as negative regulators of disease resistance in *Arabidopsis* [42]. WRKY70 was induced by CTV-B6/CaLas-B232 together, and also by CTV-B2, CTV-B6 and CaLas-B232 alone [30, 54] and in fruit infected with CaLas [55]. These results imply that WRKY70 plays a pivotal role in defense responses to both CaLas and CTV. WRKY33 was induced as a key regulator in modulating hormonal and metabolic responses to *Botrytis cinerea* infection in *Arabidopsis* [6], but the expression of WRKY33 was strongly repressed by CTV-B2/CaLas-B232 and CTV-B6/CaLas-B232, while slightly induced by CaLas-B232. This is consistent with different responses to necrotrophic (*B. cinerea*) and biotrophic (CaLas) pathogens.

Ubiquitin ligases add ubiquitin to proteins to direct them to the proteasome for degradation. Expression of *PUB22* (U-box 22) and *PUB24* encoding ubiquitin ligases were sharply down-regulated by both CTV-B2/CaLas-B232 and CTV-B6/CaLas-B232 (Additional file 3: Table S1 and Additional file 4: Table S2). This suggests that the subset of proteins whose degradation is regulated by these ubiquitin ligases would accumulate to abnormally high levels in plants infected by CTV and CaLas.

ERF1 (Ethylene response factor 1) is a positive regulator mediating the jasmonate and ethylene signaling pathways [51] and many other ERF members also play important functions in defense responses in *Arabidopsis* [58]. The expression of ERF1 was repressed in response to both CTV-B2/CaLas-B232 and CTV-B6/CaLas-B232, whereas ERF1 was induced by CTV-B6 and CaLas-B232 and no change was found in response to CTV-B2 when the pathogens were inoculated individually. The gene that encodes jasmonate-ZIM-domain protein1 (JAZ1/TIF10A) was strongly down-regulated by CTV-B2/CaLas-B232 and CTV-B6/CaLas-B232 (Additional file 3: Table S1 and Additional file 4: Table S2) whereas was up-regulated in response to CaLas-B232 alone. JAZ proteins act as repressors of JA-signaling and interact with JIN1/MYC2 [12]. A reduction of JAZ protein releases transcription factor MYC2 and further stimulates the expression of JA-responsive genes [12, 77, 81]. The expression of *JAZ1/TIFY10A* may be inducible by auxin and crosstalk between auxin and JA signaling [36].

ABA is also an important hormone associated with plant defense responses. NCEDs (nine-cis-epoxycarotenoid dioxygenase 3) are involved in the biosynthesis of ABA and their up- or down-regulation is correlated with levels of ABA. The expression of NCED3, NCED4 and NCED5 was repressed by CTV-B2/CaLas-B232 and CTV-B6/CaLas-B232 together, while NCED3 and NCED4 were over expressed in response to CTV-B6 and CaLas-B232, respectively. ABA plays a central role in plant-pathogen interactions via crosstalk with JA-SA mediated signaling pathways [27].

Auxin related genes were differentially expressed in response to CTV-B2/CaLas-B232, CTV-B6/CaLas-B232 together, and to CaLas-B232 alone, but not by either strain of CTV alone. The auxin-responsive gene *GH3.1* (*Gretchen Hagen3*) was highly repressed by both CTV-B2/CaLas-B232 and CTV-B6/CaLas-B232 (Additional file 3: Table S1 and Additional file 4: Table S2). *GH3.5* regulates both SA and auxin signaling against infection by *Pseudomonas* [90]. Over expression of *GH3.8* reinforced resistance of rice against *Xanthomonas oryzae pv. oryzae* (*Xoo*) and reduced levels of SA and JA and expression of genes responsive to SA and JA [19], demonstrating a link between the auxin responsive pathway and the SA-JA-ET signaling network in plants. *GH3.1* and *GH3.6* were strongly repressed by CTV-B2/CaLas-B232 and CTV-B6/CaLas-B232, consistent with down-regulation of auxin responsive genes in young leaves [53], but *GH3.6* was slightly induced in response to CaLas-B232 alone [30]. The repression of auxin signaling may be part of the plant immune response and could potentially enhance resistance of plants to pathogens [62, 79].

Brassinosteroids (BRs) are a unique class of plant hormones involved in various developmental processes including cell division, elongation, flowering, senescence, fruit ripening and abiotic/biotic stress responses. The BRs pathways were up regulated by either strain of CTV in conjunction with CaLas, but not by any of the pathogens separately. BR increased rice resistance to *Xanthomonas oryzae* infection [61]. The titer of CaLas was reduced by treatment with BRs, providing a new insight for the control of HLB [11].

Plants often face multiple pests and pathogens in the natural environment and consequently they apply comprehensive regulatory mechanisms to activate defense responses against various pathogens. Signaling networks and crosstalk among plant hormone mediated defense pathways played more profound and complicated roles in response to co-infection with CTV-B2/CaLas-B232 and CTV-B6/CaLas-B232 than to single infection with CTV-B2, CTV-B6 or CaLas-B232. The expression patterns of some genes related to plant hormones, such as *LOX2*, *WRKY33*, *ERF1*, *JAZ1* (Additional file 3: Table S1 and Additional file 4: Table S2), in response to dual pathogen infection were opposite to the response observed with single infections by the same pathogens.

The composition of ribosomal subunits was especially affected by co-infection of CaLas-B232 and CTV-B6, in contrast with infection by the same pathogens in single pathogen infections. The down-regulation of ribosomal biogenesis was also found in both roots and leaves from Fe-deficient *Arabidopsis* [70]. PSI and PSII are the largest sinks for Fe and majority of Fe is stored in chloroplasts. Decreased photosynthetic performances as well as tetrapyrrole biosynthesis are hallmarks of Fe deficiency in plants [70]. The repression of ribosomal biogenesis we observed could also result from Fe deficiency in the leaves we sampled, particularly when the hosts are infected with both CTV-B6 and CaLas together [70]. A failure to properly recalibrate cellular Fe homeostasis may be responsible for the decrease of ribosomal subunits. This repression is highly organ-specific, rather than general [70, 84].

## Conclusion

Carbohydrate, amino acid and lipid metabolism were deeply reconfigured in response to CTV-B2/CaLas-B232 and CTV-B6/CaLas-B232, much more so than in response to infection by any of the pathogens alone. The photosynthetic, glycolytic, oxidative pentose phosphate and tricarboxylic acid pathways were all repressed by CTV-B2/CaLas-B232 and especially by CTV-B6/CaLas-B232. The repression of these energy generating processes limited plant growth and defense responses [7]. The circadian system was also strongly perturbed by both double and single pathogen infection. Defense pathways, including those linked to the hormones jasmonic acid, salicylic acid, gibberellic acid, abscisic acid, auxin, brassinosteroids and cytokinins were all perturbed by CTV-B6/CaLas-B232, and to a lesser extent by CTV-B2/CaLas-B232, CaLas-B232, CTV-B6 and CTV-B2. Transport systems were also heavily perturbed and many transporters were activated. ZIP1, ZIP4, ZIP5 and PP2-B15 showed very different expression patterns in response to double pathogen infection compared to single pathogen infection. CTV-B6 may cooperate with CaLas-B232 in weakening the plant through root decline and phloem blockage. Defense responses activated by the mild strain CTV-B2 may provide some beneficial effects against CaLas-B232. Although many host genes were expressed differently in response to double-infections as compared to single infections with the same pathogens, the symptoms of CaLas and CTV-B6 infection were produced. The interaction of the pathogens within the host may lead to a better or worse result for the host plant.

## Methods

### Inoculation of experimental trees with CTV and CaLas

CTV mild strain B2 (T30 genotype, Florida), severe strain B6 (Complex genotype, SY568, California) and CaLas strain B232 (Thailand) are maintained *in planta* as part of the Exotic Pathogens of Citrus Collection (EPCC) at the USDA-ARS Beltsville Agricultural Research Center (BARC) in Beltsville, MD. Experimental seedling trees were ‘Valencia’ sweet orange. CaLas-B232 was inoculated by bud grafting into two groups of 10 trees. Three months later, one group of the trees was inoculated with CTV-B2 and another group was inoculated with CTV-B6. Ten trees were mock-inoculated with healthy buds as controls.

### RNA preparation and qPCR

DNA and RNA extractions and RT-PCR and qPCR testing for the presence of pathogens were performed as previously described [30]. Three months after the CTV inoculations, and therefore 6 months after the CaLas inoculations, trees that tested positive for both CTV-B2 and CaLas-B232 (CTV-B2/CaLas-B232) or both CTV-B6 and CaLas-B232 (CTV-B6/CaLas-B232) by PCR were selected for analysis. Three to five young, soft, not fully expanded leaves of uniform size and without symptoms were collected, frozen in liquid nitrogen and kept at −80 °C. RNA was extracted as described [30] and assessed for quantity and quality with a Qubit 2.0 Fluorometer (Invitrogen) and Bioanalyzer 2100 (Agilent Technologies, Santa Clara, CA). Nine RNA samples (RIN ≥ 7.5), comprised of three RNA replicates, each from individual trees for each treatment (CTV-B2/CaLas-B232-infected, CTV-B6/CaLas-B232-infected and healthy citrus) were sent to Otogenetics (Norcross, GA, USA) for paired-end sequencing with the Illumina HiSeq 2500 platform.

### Statistical analyses of RNA sequence libraries

Libraries of paired-end reads (2 × 100 nucleotides) for the nine citrus RNA extracts were generated with the Illumina HiSeq2500. After removing low-quality reads and adaptor sequences from the raw reads, high-quality reads were obtained and mapped to the *C. sinensis* reference genome [85] with Bowtie [46]. Expression levels of transcripts (log_2_ fold change) were calculated with DESeq2 [53]. Sequence similarity comparisons of the sequences identified in the reference genome of *C. sinensis* were made with both *Arabidopsis thaliana* and another *C. sinensis* genome [32]. Differentially expressed transcripts (DETs) were filtered with cut-off values, Padj ≤0.1, |log_2_FC| ≥ 1 and e-value ≤ e^−5^. DETs were enriched by Panther [59] with corresponding *Arabidopsis* orthologs and further assigned to metabolic pathways by reference to the Kyoto Encyclopedia of Genes and Genomes (KEGG) and functionally classified by Mapman [80].

### RT-qPCR analysis

A total of 4 μg of RNA was reverse transcribed for first-strand cDNA syntheses using GoTaq® 2-step RT-qPCR system (Promega, Madison, WI) according to the manufacturer’s instructions. cDNA was diluted three fold with 1 X TE and stored at −20 °C for use. Gene-specific primers (Additional file 5: Table S3) were designed with an online tool (Integrated DNA Technologies, Coralville, IA) with melting temperatures of 60 °C ± 5 °C. qPCR reactions were performed with GoTaq® qPCR Master Mix (Promega) in a Bio-Rad CFX96 system. Twenty microliters of reaction mixture was added to each well with three replicated plant samples for each primer pair for each sample. The reaction program was set at 95 °C for 3 min, and 40 cycles of 95 °C for 10 s, 60 °C for 30 s and 72 °C for 30 s. Melting curves were analyzed to ensure that a single product was amplified. *Actin* was used as the internal control. The *Actin* gene has been verified to be relatively stable within the large number of potential references genes [48, 87]; (Additional file 10: Figure S7). The 2^-△△Ct^ method was applied for relative quantification of gene expression [50].

## Additional files

Additional file 1: Figure S1. Summary of reads from *Citrus sinensis* infected with CTV-B2/CaLas-B232 or CTV-B6/CaLas-B232. Reads were obtained through paired-end RNA sequencing and mapped to the *C. sinensis* genome. **HC**, Self-inoculated healthy control. Bars indicate the standard error of the mean of three replicates. (PDF 88 kb)

Additional file 2: Figure S2. Differentially expressed transcripts in diseased vs. healthy *Citrus sinensis*. Transcripts were up- or down-regulated in response to co-infection by CTV-B2/CaLas-B232 or CTV-B6/CaLas-B232. **HC**, Self-inoculated healthy control. (PDF 103 kb)

Additional file 3: Table S1. Complete differentially expressed transcripts (Log2 fold-change) in response to dual and single infection by CTV-B2 and CaLas-B232. (XLSX 140 kb)

Additional file 4: Table S2. Complete differentially expressed transcripts (Log2 fold-change) in response to dual and single infection by CTV-B6 and CaLas-B232 (XLSX 286 kb)

Additional file 5: Table S3. Primers used for qRT-PCR validation of differentially expressed transcripts (XLSX 17 kb)

Additional file 6: Figure S3. Comparison of RT-qPCR and RNA-Seq estimates of gene expression in diseased sweet orange. X-axis: Abbreviation of gene names (see text for details); Y-axis: Log_2_ Fold-change in expression. Error bars show the standard error of three biological replicates for each treatment. (PDF 219 kb)

Additional file 7: Figure S4. Effects on tetrapyrrole biosynthetic pathway by co-infection with CTV-B2/CaLas-B232 and CTV-B6/CaLas-B232. (PDF 124 kb)

Additional file 8: Figure S5. Typical leaf symptoms caused by CTV-B2, CTV-B6 or CaLas-B232 in single infection. **A**, no symptoms caused by CTV-B2; **B** and **C**, chlorosis caused by CaLas-B232; **D**, **E** and **F**, chlorosis, leaf curing and vein corking symptoms caused by CTV-B6. (PDF 263 kb)

Additional file 9: Figure S6. Plant hormone pathways affected by co-infection with CTV-B2/CaLas-B232 and CTV-B6/CaLas-B232. Red boxes, down-regulated genes; Green boxes, up-regulated genes. (PDF 230 kb)

Additional file 10: Figure S7. Expression of the actin gene estimated by RT-qPCR. HC, healthy control; B2/232, CTV-B2/CaLas-B232; B6/232, CTV-B6/CaLas-B232. Y-axis, Cq value. Bars with standard error of the mean expression level of the actin gene with three technical replicates for each biological replicate. (PDF 56 kb)

## Abbreviations

CqaLas: ‘*Candidatus* Liberibacter asiaticus’
CTV: *Citrus tristeza virus*
DEG: Differentially expressed gene; GO: Gene Ontology; FC: fold change; BP: biological process; TF: transcription factor
DET: Differentially expressed transcript
HLB: Huanglongbing

## References

[1] Abadía J, Morales F, Abadía A (1999). Photosystem II efficiency in low chlorophyll, iron-deficient leaves. Plant Soil.

[2] Albrecht U, Bowman KD (2008). Gene expression in *Citrus sinensis* (L.) Osbeck following infection with the bacterial pathogen *Candidatus* Liberibacter asiaticus causing Huanglongbing in Florida. Plant Sci.

[3] Albrecht U, Bowman KD (2012). Transcriptional response of susceptible and tolerant citrus to infection with *Candidatus* Liberibacter asiaticus. Plant Sci.

[4] Andrés-Colás N, Sancenón V, Rodríguez-Navarro S, Mayo S, Thiele DJ, Ecker JR (2006). The *Arabidopsis* heavy metal P-type ATPase HMA5 interacts with metallochaperones and functions in copper detoxification of roots. Plant J.

[5] Aritua V, Achor D, Gmitter FG, Albrigo G, Wang N (2013). Transcriptional and microscopic analyses of citrus stem and root responses to *Candidatus* Liberibacter asiaticus infection. PLoS One.

[6] Birkenbihl RP, Diezel C, Somssich IE (2012). *Arabidopsis* WRKY33 is a key transcriptional regulator of hormonal and metabolic responses toward *Botrytis cinerea* infection. Plant Physiol.

[7] Bolton MD (2009). Primary metabolism and plant defense-fuel for the fire. Mol Plant-Microbe Interact.

[8] Bové JM (2006). Huanglongbing: a destructive, newly-emerging, century old disease of citrus. J Plant Pathol.

[9] Bové JM, Renaudin J, Saillard C, Foissac X, Garnier M (2003). *Spiroplasma citri*, a plant pathogenic mollicute: relationships with its two hosts, the plant and insect vector. Annu Rev Phytopathol.

[10] Bowman K, Albrecht U. Comparison of gene expression changes in susceptible, tolerant and resistant hosts in response to infection with *Citrus tristeza virus* and huanglongbing. J Citrus Pathol. 2015;2(1). http://escholarship.org/uc/item/5qt4z9c0#page-4.

[11] Canales E, Coll Y, Hernández I, Portieles R, García MR (2016). *Candidatus* Liberibacter asiaticus’, causal agent of citrus huanglongbing, is reduced by treatment with brassinosteroids. PLoS One.

[12] Chini A, Fonseca S, Fernandez G, Adie B, Chico J, Lorenzo O (2007). The JAZ family of repressors is the missing link in jasmonate signalling. Nature.

[13] Chinnappa KSA, Nguyen TTS, Hou J, Wu Y, McCurdy DW (2013). Phloem parenchyma transfer cells in *Arabidopsis-*an experimental system to identify transcriptional regulators of wall ingrowth formation. Front Plant Sci.

[14] Cristofani-Yaly M, Berger IJ, Targon MLP, Takita MA, Dorta S, Freitas-Astúa J (2007). Differential expression of genes identified from Poncirus Trifoliata tissue inoculated with CTV through EST analysis and in silico hybridization. Genet Mol Biol.

[15] Dalchau N, Baek SJ, Briggs HM, Robertson FC, Dodd AN, Gardner MJ (2011). The circadian oscillator gene GIGANTEA mediates a long-term response of the *Arabidopsis thaliana* circadian clock to sucrose. PNAS USA..

[16] Dawson WO, Bar-Joseph M, Garnsey SM, Moreno P (2015). *Citrus Tristeza Virus*: making an ally from an enemy. Annu Rev Phytopathol.

[17] De Bruyne L, Höfte M, De Vleesschauwer D (2014). Connecting growth and defense: the emerging roles of brassinosteroids and gibberellins in plant innate immunity. Mol Plant.

[18] Deeken R, Geiger D, Fromm J, Koroleva O, Ache P, Langenfeld-Heyser R (2002). Loss of the AKT2/3 potassium channel affects sugar loading into the phloem of *Arabidopsis*. Planta.

[19] Ding X, Cao Y, Huang L, Zhao J, Xu C, Li, X et al. Activation of the indole-3-acetic acid–amido synthetase GH3–8 suppresses expansin expression and promotes salicylate-and jasmonate-independent basal immunity in rice. Plant Cell 2008;20(1): 228-240. 10.1105/tpc.107.055657 PMC2254934 18192436

[20] Doering-Saad C, Newbury H, Couldridge C, Bale J, Pritchard J (2006). A phloem-enriched cDNA library from *Ricinus*: insights into phloem function. J Exp Botany.

[21] Doering-Saad C, Newbury HJ, Bale JS, Pritchard J (2002). Use of aphid stylectomy and RT-PCR for the detection of transporter mRNAs in sieve elements. J Exp Botany.

[22] Duan Y, Zhou L, Hall DG, Li W, Doddapaneni H, Lin H (2009). Complete genome sequence of citrus huanglongbing bacterium, ‘*Candidatus* Liberibacter asiaticus’ obtained through metagenomics. Mol Plant-Microbe Interact.

[23] Edwards J, Martin AP, Andriunas F, Offler CE., Patrick JW., McCurdy, DW. GIGANTEA is a component of a regulatory pathway determining wall ingrowth deposition in phloem parenchyma transfer cells of Arabidopsis Thaliana. Plant J 2010;63(4): 651-661. 10.1111/j.1365-313X.2010.04269.x 20545890

[24] Fan J, Chen C, Achor DS, Brlansky RH, Li ZG, Gmitter FG (2013). Differential anatomical responses of tolerant and susceptible citrus species to the infection of ‘*Candidatus* Liberibacter asiaticus. Physiol Mol Plant Path.

[25] Fan J, Chen C, Yu Q, Brlansky RH, Lia ZG, Gmitter FG (2011). Comparative iTRAQ proteome and transcriptome analyses of sweet orange infected by “*Candidatus* Liberibacter asiaticus”. Physiol Plant.

[26] Fan J, Chen C, Yu Q, Khalaf A, Achor DS, Brlansky RH (2012). Comparative transcriptional and anatomical analyses of tolerant rough lemon and susceptible sweet orange in response to ‘*Candidatus* Liberibacter asiaticus’ infection. Mol Plant-Microbe Interact.

[27] Fan J, Hill L, Crooks C, Doerner P, Lamb C (2009). Abscisic acid has a key role in modulating diverse plant-pathogen interactions. Plant Physiol.

[28] Fischer WN, Loo DD, Ludewig U, Boorer KJ, Tegeder M, Rentsch D (2002). Low and high affinity amino acid H+−cotransporters for cellular import of neutral and charged amino acids. Plant J.

[29] Folimonova SY, Achor DS (2010). Early events of citrus greening (huanglongbing) disease development at the ultrastructural level. Phytopathology.

[30] Fu S, Shao J, Zhou C, Hartung JS (2016). Transcriptome analysis of sweet orange trees infected with ‘*Candidatus* Liberibacter asiaticus’ and two strains of *Citrus Tristeza Virus*. BMC Genomics.

[31] Gao W, Xiao S, Li HY, Tsao SW, Chye ML (2009). *Arabidopsis thaliana* acyl-CoA-binding protein ACBP2 interacts with heavy metal binding farnesylated protein AtFP6. New Phytol.

[32] Gmitter Jr. FG, Chen C, Machado MA, de Souza AA, Ollitrault P., Froehlicher Y et al. Citrus genomics. Tree Genet Genomes. 2012; 8(3): 611-626.

[33] Goodspeed D, Chehab EW, Min-Venditti A, Braam J, Covington MF (2012). *Arabidopsis* synchronizes jasmonate-mediated defense with insect circadian behavior. PNAS USA.

[34] Gould N, Thorpe MR, Minchin PE, Pritchard J, White PJ (2004). Solute is imported to elongating root cells of barley as a pressure driven-flow of solution. Funct. Plant Biol.

[35] Graham JH, Johnson EG, Gottwald TR, Irey MS (2013). Presymptomatic fibrous root decline in citrus trees caused by Huanglongbing and potential interaction with *Phytophthora* spp. Plant Dis.

[36] Grunewald W, Vanholme B, Pauwels L, Plovie E, Inzé D, Gheysen G (2009). Expression of the *Arabidopsis* jasmonate signalling repressor JAZ1/TIFY10A is stimulated by auxin. EMBO Rep.

[37] Harmer SL (2009). The circadian system in higher plants. Annu Rev Plant Biol.

[38] Hartung JS, Shao J, Kuykendall LD (2011). Comparison of the ‘*Ca*. Liberibacter asiaticus’ genome adapted for an intracellular lifestyle with other members of the Rhizobiales. PLoS One.

[39] Hunt E, Gattolin S, Newbury HJ, Bale JS, Tseng H-M, Barrett DA (2010). A mutation in amino acid permease AAP6 reduces the amino acid content of the *Arabidopsis* sieve elements but leaves aphid herbivores unaffected. J exp. Botany.

[40] Jiang C-J, Shimono M, Sugano S, Kojima M, Liu X, Inoue H (2013). Cytokinins act synergistically with salicylic acid to activate defense gene expression in rice. Mol Plant-Microbe Interact.

[41] Johnson EG, Wu J, Bright DB, Graham JH (2014). Association of ‘*Candidatus* Liberibacter asiaticus’ root infection, but not phloem plugging with root loss on huanglongbing-affected trees prior to appearance of foliar symptoms. Plant Pathol.

[42] Journot-Catalino N, Somssich IE, Roby D, Kroj T (2006). The transcription factors WRKY11 and WRKY17 act as negative regulators of basal resistance in *Arabidopsis thaliana*. Plant Cell.

[43] Karley A, Douglas A, Parker W (2002). Amino acid composition and nutritional quality of potato leaf phloem sap for aphids. J Exp Botany.

[44] Kehr J, Butz A. Endogenous RNA constituents of the phloem and their possible roles in long-distance signaling in phloem. In: Thompson GA, van Bel AJE, editors. Molecular cell biology, systemic communication, biotic interactions. Oxford,UK: Wiley-Blackwell. 10.1002/9781118382806.ch95.

[45] Kim JS, Sagaram US, Burns JK, Li JL, Wang N (2009). Response of sweet orange (*Citrus sinensis*) to ‘*Candidatus* Liberibacter asiaticus’ infection: microscopy and microarray analyses. Phytopathology.

[46] Langmead B, Trapnell C, Pop M, Salzberg SL. Ultrafast and memory-efficient alignment of short DNA sequences to the human genome. Genome Biol. 2009;10 (R25). https://genomebiology.biomedcentral.com/track/pdf/10.1186/gb-2009-10-3-r25?site=genomebiology.biomedcentral.com. 10.1186/gb-2009-10-3-r25 PMC2690996 19261174

[47] Liao HL, Burns JK (2012). Gene expression in *Citrus sinensis* fruit tissues harvested from huanglongbing-infected trees: comparison with girdled fruit. J Exp Botany.

[48] Liu Q, Zhu A, Chai L, Zhou W, Yu K, Ding J (2009). Transcriptome analysis of a spontaneous mutant in sweet orange [*Citrus sinensis* (L.) Osbeck] during fruit development. J Exp Botany.

[49] Liu Y, Wang G, Wang Z, Yang F, Wu G, Hong N (2012). Identification of differentially expressed genes in response to infection of a mild *Citrus tristeza virus* isolate in *Citrus aurantifolia* by suppression subtractive hybridization. Sci Hort.

[50] Livak KJ, Schmittgen TD (2001). Analysis of relative gene expression data using real-time quantitative PCR and the 2^-ΔΔCT^ method. Methods.

[51] Lorenzo O, Piqueras R, Sánchez-Serrano JJ, Solano R (2003). ETHYLENE RESPONSE FACTOR1 integrates signals from ethylene and jasmonate pathways in plant defense. Plant Cell.

[52] Lough TJ, Lucas WJ (2006). Integrative plant biology: role of phloem long-distance macromolecular trafficking. Annu Rev Plant Biol.

[53] Love MI, Huber W, Anders S (2014). Moderated estimation of fold change and dispersion for RNA-seq data with DESeq2. Genome Biol.

[54] Mafra V, Martins P, Francisco C, Ribeiro-Alves M, Freitas-Astua J, Machado MA (2013). *Candidatus* Liberibacter americanus induces significant reprogramming of the transcriptome of the susceptible citrus genotype. BMC Genomics.

[55] Martinelli F, Reagan RL, Uratsu SL, Phu ML, Albrecht U, Zhao W (2013). Gene regulatory networks elucidating huanglongbing disease mechanisms. PLoS One.

[56] Martinelli F, Uratsu SL, Albrecht U, Reagan RL, Phu ML, Britton M (2012). Transcriptome profiling of citrus fruit response to Huanglongbing disease. PLoS One.

[57] Mauch-Mani B, Mauch F (2005). The role of abscisic acid in plant–pathogen interactions. Curr Opin Plant Biol.

[58] McGrath KC, Dombrecht B, Manners JM, Schenk PM, Edgar CI, Maclean DJ (2005). Repressor-and activator-type ethylene response factors functioning in jasmonate signaling and disease resistance identified via a genome-wide screen of *Arabidopsis* transcription factor gene expression. Plant Physiol.

[59] Mi H, Dong Q, Muruganujan A, Gaudet P, Lewis S, Thomas PD (2010). PANTHER version 7: improved phylogenetic trees, orthologs and collaboration with the gene ontology consortium. Nucleic Acid Res.

[60] Moreno P, Ambros S, Albiach-Marti MR, Guerri J, Pena L (2008). *Citrus tristeza virus* : a pathogen that changed the course of the citrus industry. Mol Plant Pathol.

[61] Nakashita H, Yasuda M, Nitta T, Asami T, Fujioka S, Arai Y (2003). Brassinosteroid functions in a broad range of disease resistance in tobacco and rice. Plant J.

[62] Navarro L, Dunoyer P, Jay F, Arnold B, Dharmasiri N, Estelle M (2006). A plant miRNA contributes to antibacterial resistance by repressing auxin signaling. Science.

[63] Okumoto S, Schmidt R, Tegeder M, Fischer WN, Rentsch D, Frommer WB (2002). High affinity amino acid transporters specifically expressed in xylem parenchyma and developing seeds of Arabidopsis. J Biol Chem.

[64] Park DH, Somers DE, Kim YS, Choy Y, Lim HK, Soh MS (1999). Control of circadian rhythms and photoperiodic flowering by the *Arabidopsis* GIGANTEA gene. Science.

[65] Pieterse CM, Leon-Reyes A, Van der Ent S, Van Wees SC (2009). Networking by small-molecule hormones in plant immunity. Nature Chem Biol.

[66] Ponder K, Pritchard J, Harrington R, Bale J (2000). Difficulties in location and acceptance of phloem sap combined with reduced concentration of phloem amino acids explain lowered performance of the aphid *Rhopalosiphum padi* on nitrogen deficient barley (*Hordeum vulgare*) seedlings. Entomologia Experimentalis et Applicata.

[67] Rawat N, Kiran SP, Du D, Gmitter FG, Deng Z (2015). Comprehensive meta-analysis, co-expression, and miRNA nested network analysis identifies gene candidates in citrus against Huanglongbing disease. BMC Plant Biol.

[68] Rentsch D, Hirner B, Schmelzer E, Frommer WB (1996). Salt stress-induced proline transporters and salt stress-repressed broad specificity amino acid permeases identified by suppression of a yeast amino acid permease-targeting mutant. Plant Cell.

[69] Roden LC, Ingle RA (2009). Lights, rhythms, infection: the role of light and the circadian clock in determining the outcome of plant–pathogen interactions. Plant Cell.

[70] Rodríguez-Celma J, Pan IC, Li W, Lan P, Buckhout TJ, Schmidt W. The transcriptional response of *Arabidopsis* leaves to Fe deficiency. Frontiers Plant Sci. 2013;4(276). https://www.ncbi.nlm.nih.gov/pmc/articles/PMC3719017/pdf/fpls-04-00276.pdf. 10.3389/fpls.2013.00276 PMC3719017 23888164

[71] Rosa BA, Jiao Y, Oh S, Montgomery BL, Qin W, Chen J (2012). Frequency-based time-series gene expression recomposition using PRIISM. BMC Syst Biol.

[72] Roudier F (2002). The COBRA family of putative GPI-anchored proteins in Arabidopsis. A new fellowship in expansion. Plant Physiol.

[73] Schindelman G., Benfey P. NCOBRA encodes a putative GPI-anchored protein, which is polarly localized and necessary for oriented cell expansion in *Arabidopsis.* Genes Dev. 2001; 15: 1115-1127. 10.1101/gad.879101 PMC312689 11331607

[74] Schwachtje J, Baldwin IT (2008). Why does herbivore attack reconfigure primary metabolism?. Plant Physiol.

[75] Slewinski TL, Meeley R, Braun DM (2009). Sucrose transporter1 functions in phloem loading in maize leaves. J Exp Botany.

[76] Spann TM, Schumann AW. The role of plant nutrients in disease development with emphasis on citrus and huanglongbing. Proc Florida State Hort Soc. 2009:169–71.

[77] Staswick PE (2008). JAZing up jasmonate signaling. Trends Plant Sci.

[78] Terry N, Low G (1982). Leaf chlorophyll content and its relation to the intracellular localization of iron. J Plant Nutr.

[79] Thilmony R, Underwood W, He SY (2006). Genome wide transcriptional analysis of the *Arabidopsis thaliana* interaction with the plant pathogen *Pseudomonas syringae pv. tomato* DC3000 and the human pathogen *Escherichia coli* O157: H7. Plant J.

[80] Thimm O, Bläsing O, Gibon Y, Nagel A, Meyer S, Krüger P (2004). MAPMAN: a user-driven tool to display genomics data sets onto diagrams of metabolic pathways and other biological processes. Plant J.

[81] Thines B, Katsir L, Melotto M, Niu Y, Mandaokar A, Liu G (2007). JAZ repressor proteins are targets of the SCFCOI1 complex during jasmonate signalling. Nature.

[82] Wang D, Amornsiripanitch N, Dong X (2006). A genomic approach to identify regulatory nodes in the transcriptional network of systemic acquired resistance in plants. PLoS Pathog.

[83] Wang D, Pajerowska-Mukhtar K, Culler AH, Dong X (2007). Salicylic acid inhibits pathogen growth in plants through repression of the auxin signaling pathway. Curr Biol.

[84] Wang N, Trivedi P (2013). Citrus huanglongbing: a newly relevant disease presents unprecedented challenges. Phytopathology.

[85] Xu Q, Chen L-L, Ruan X, Chen D, Zhu A, Chen C (2013). The draft genome of sweet orange (*Citrus sinensis*). Nat Genet.

[86] Yang F, Wang G-P, Jiang B, Liu Y-H, Liu Y, Wu G-W (2013). Differentially expressed genes and temporal and spatial expression of genes during interactions between Mexican lime (*Citrus aurantifolia*) and a severe *Citrus tristeza virus* isolate. Physiol Mol Plant Pathol.

[87] Yan J, Yuan F, Long G (2012). Selection of reference genes for quantitative real-time RT-PCR analysis in citrus. Mol Biol Rep.

[88] Yu J-W, Rubio V, Lee N-Y, Bai S, Lee S-Y, Kim S-S (2008). COP1 and ELF3 control circadian function and photoperiodic flowering by regulating GI stability. Mol Cell.

[89] Zeier J (2013). New insights into the regulation of plant immunity by amino acid metabolic pathways. Plant Cell Environ.

[90] Zhang Z, Li Q, Li Z, Staswick PE, Wang M-Y, Zhu Y (2007). Dual regulation role of GH3. 5 in salicylic acid and auxin signaling during *Arabidopsis-Pseudomonas syringae* interaction. Plant Physiol.

[91] Zheng Z-L, Zhao Y (2013). Transcriptome comparison and gene coexpression network analysis provide a systems view of citrus response to ‘*Candidatus* Liberibacter asiaticus’ infection. BMC Genomics.

[92] Zhong Y, Cheng C-Z, Jiang N-H, Jiang B, Zhang Y-Y, Wu B (2015). Comparative Transcriptome and iTRAQ proteome analyses of citrus root responses to *Candidatus* Liberibacter asiaticus infection. PLoS One.

