# Supplementary material for: Transcriptional analysis of sweet orange trees co-infected with ‘Candidatus Liberibacter asiaticus’ and mild or severe strains of Citrus tristeza virus
Source: BMC Genomics. 2017 Oct 31;18:837. doi: 10.1186/s12864-017-4174-8 (PMC5664567; doi:10.1186/s12864-017-4174-8)
Supplement: Supplementary file 1 — Summary of reads from Citrus sinensis infected with CTV-B2/CaLas-B232 or CTV-B6/CaLas-B232. Reads were obtained through paired-end RNA sequencing and mapped to the C. sinensis genome. HC, Self-inoculated healthy control. Bars indicate the standard error of the mean of three replicates. (PDF 88 kb) [file 12864_2017_4174_MOESM1_ESM.pdf]

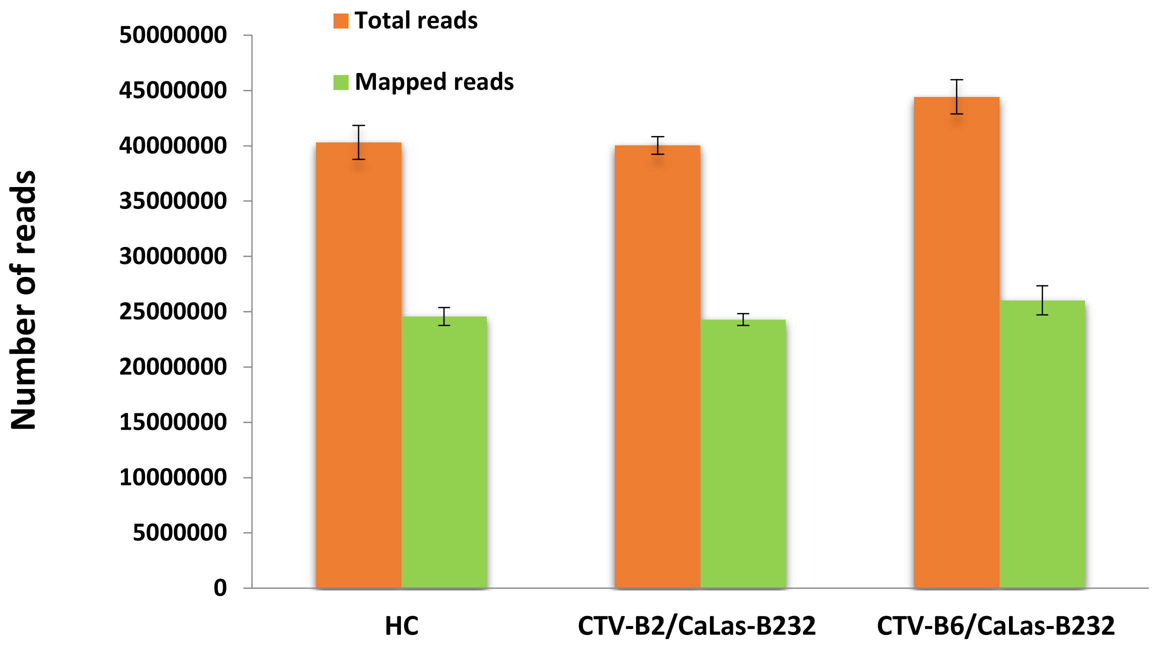

Figure S1. Summary of reads from *Citrus sinensis* infected with CTV-B2/CaLas-B232 or CTV-B6/CaLas-B232. Reads were obtained through paired-end RNA sequencing and mapped to the *C. sinensis* genome. **HC**, Self-inoculated healthy control. Bars indicate the standard error of the mean of three replicates.
