# Supplementary material for: Transcriptional analysis of sweet orange trees co-infected with ‘Candidatus Liberibacter asiaticus’ and mild or severe strains of Citrus tristeza virus
Source: BMC Genomics. 2017 Oct 31;18:837. doi: 10.1186/s12864-017-4174-8 (PMC5664567; doi:10.1186/s12864-017-4174-8)
Supplement: Supplementary file 2 — Differentially expressed transcripts in diseased vs. healthy Citrus sinensis. Transcripts were up- or down-regulated in response to co-infection by CTV-B2/CaLas-B232 or CTV-B6/CaLas-B232. HC, Self-inoculated healthy control. (PDF 103 kb) [file 12864_2017_4174_MOESM2_ESM.pdf]

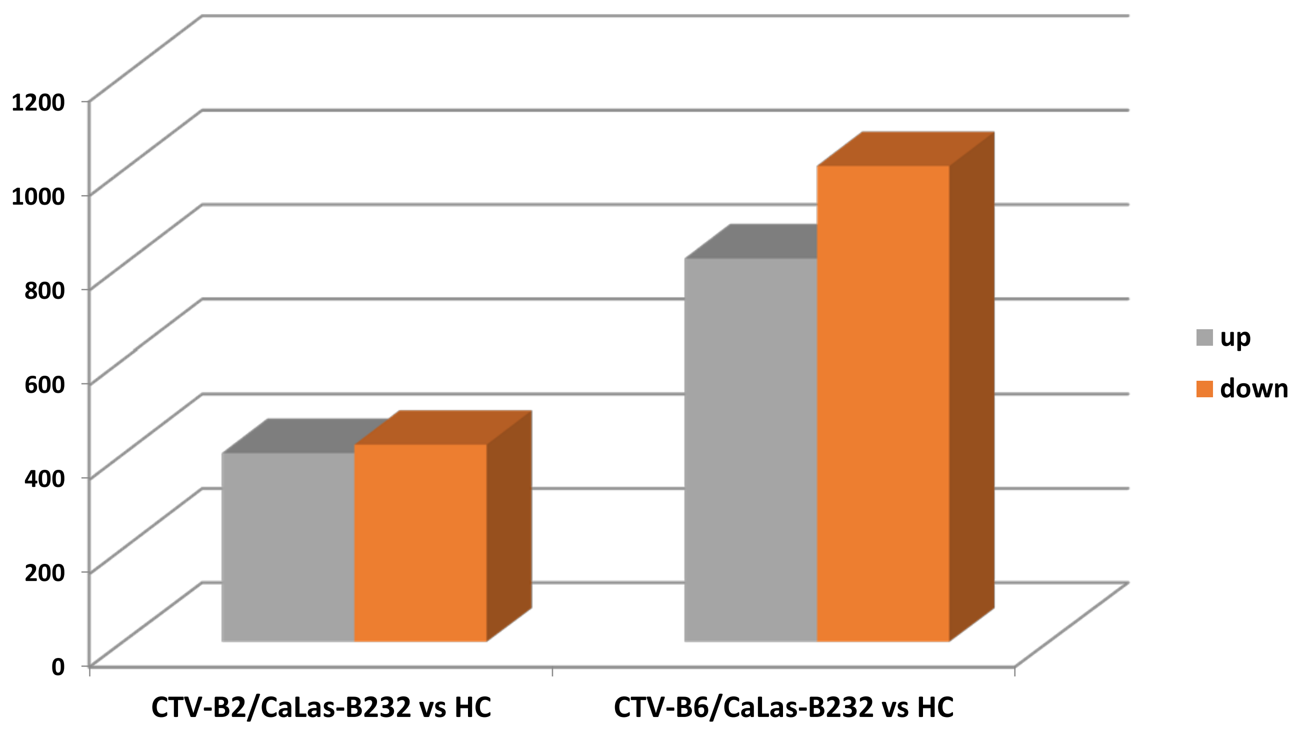

Figure S2. Differentially expressed transcripts in diseased vs. healthy *Citrus sinensis*. Transcripts were up- or down-regulated in response to co-infection by CTV-B2/CaLas-B232 or CTV-B6/CaLas-B232. **HC**, Self-inoculated healthy control.
