# Supplementary material for: Transcriptional analysis of sweet orange trees co-infected with ‘Candidatus Liberibacter asiaticus’ and mild or severe strains of Citrus tristeza virus
Source: BMC Genomics. 2017 Oct 31;18:837. doi: 10.1186/s12864-017-4174-8 (PMC5664567; doi:10.1186/s12864-017-4174-8)
Supplement: Supplementary file 6 — Comparison of RT-qPCR and RNA-Seq estimates of gene expression in diseased sweet orange. X-axis: Abbreviation of gene names (see text for details); Y-axis: Log2 Fold-change in expression. Error bars show the standard error of three biological replicates for each treatment. (PDF 219 kb) [file 12864_2017_4174_MOESM6_ESM.pdf]

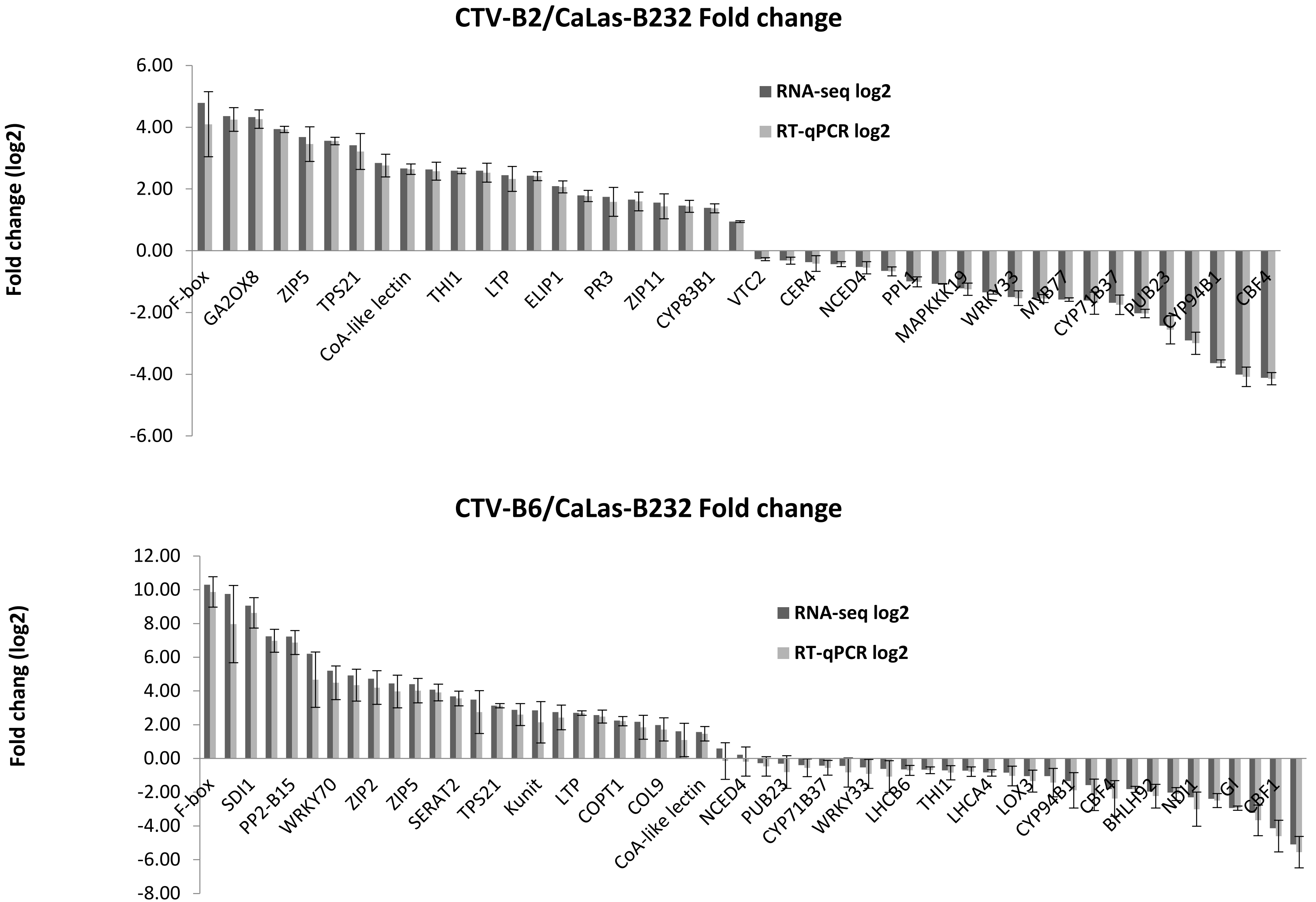

Figure S3. Comparison of RT-qPCR and RNA-Seq estimates of gene expression in diseased sweet orange. X-axis: Abbreviation of gene names (see text for details); Y-axis: Fold change in expression. Error bars show the standard error of three biological replicates for each treatment.
