# Supplementary material for: Transcriptional analysis of sweet orange trees co-infected with ‘Candidatus Liberibacter asiaticus’ and mild or severe strains of Citrus tristeza virus
Source: BMC Genomics. 2017 Oct 31;18:837. doi: 10.1186/s12864-017-4174-8 (PMC5664567; doi:10.1186/s12864-017-4174-8)
Supplement: Supplementary file 7 — Effects on tetrapyrrole biosynthetic pathway by co-infection with CTV-B2/CaLas-B232 and CTV-B6/CaLas-B232. (PDF 124 kb) [file 12864_2017_4174_MOESM7_ESM.pdf]

CTV-B2/CaLas-B232

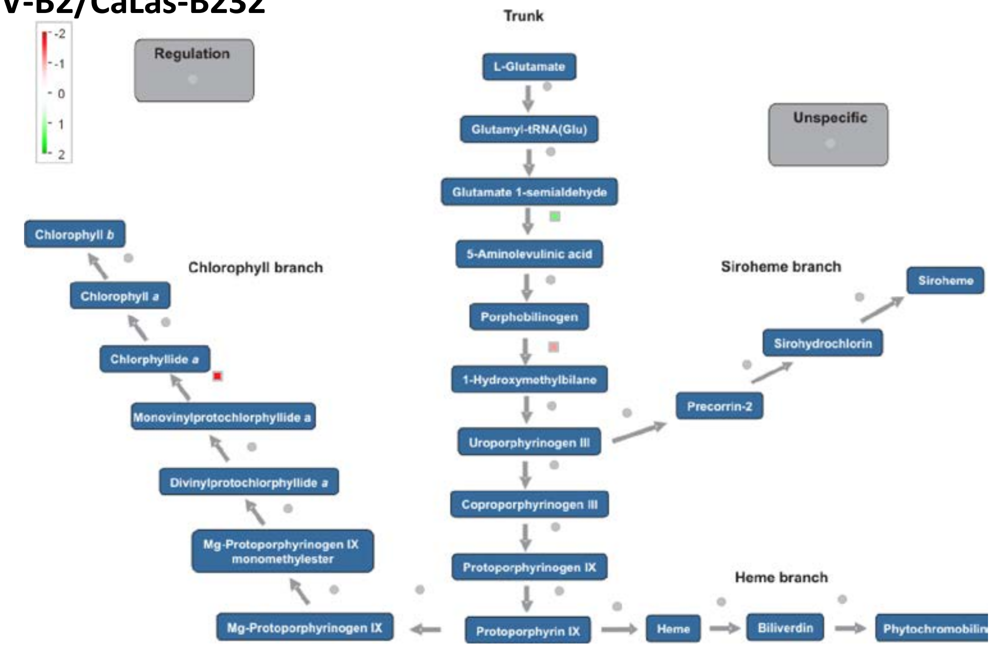

CTV-B6/CaLas-B232

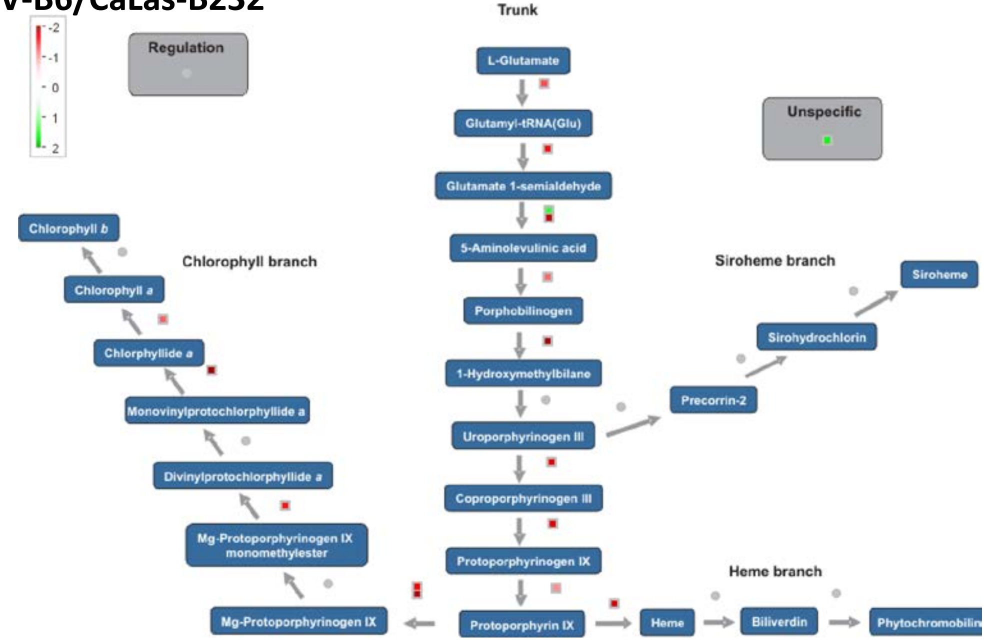

Figure S4. Effects on tetrapyrrole biosynthetic pathway by co-infection with CTV-B2/CaLas-B232 and CTV-B6/CaLas-B232.
