# Supplementary material for: Transcriptional analysis of sweet orange trees co-infected with ‘Candidatus Liberibacter asiaticus’ and mild or severe strains of Citrus tristeza virus
Source: BMC Genomics. 2017 Oct 31;18:837. doi: 10.1186/s12864-017-4174-8 (PMC5664567; doi:10.1186/s12864-017-4174-8)
Supplement: Supplementary file 8 — Typical leaf symptoms caused by CTV-B2, CTV-B6 or CaLas-B232 in single infection. A, no symptoms caused by CTV-B2; B and C, chlorosis caused by CaLas-B232; D, E and F, chlorosis, leaf curing and vein corking symptoms caused by CTV-B6. (PDF 263 kb) [file 12864_2017_4174_MOESM8_ESM.pdf]

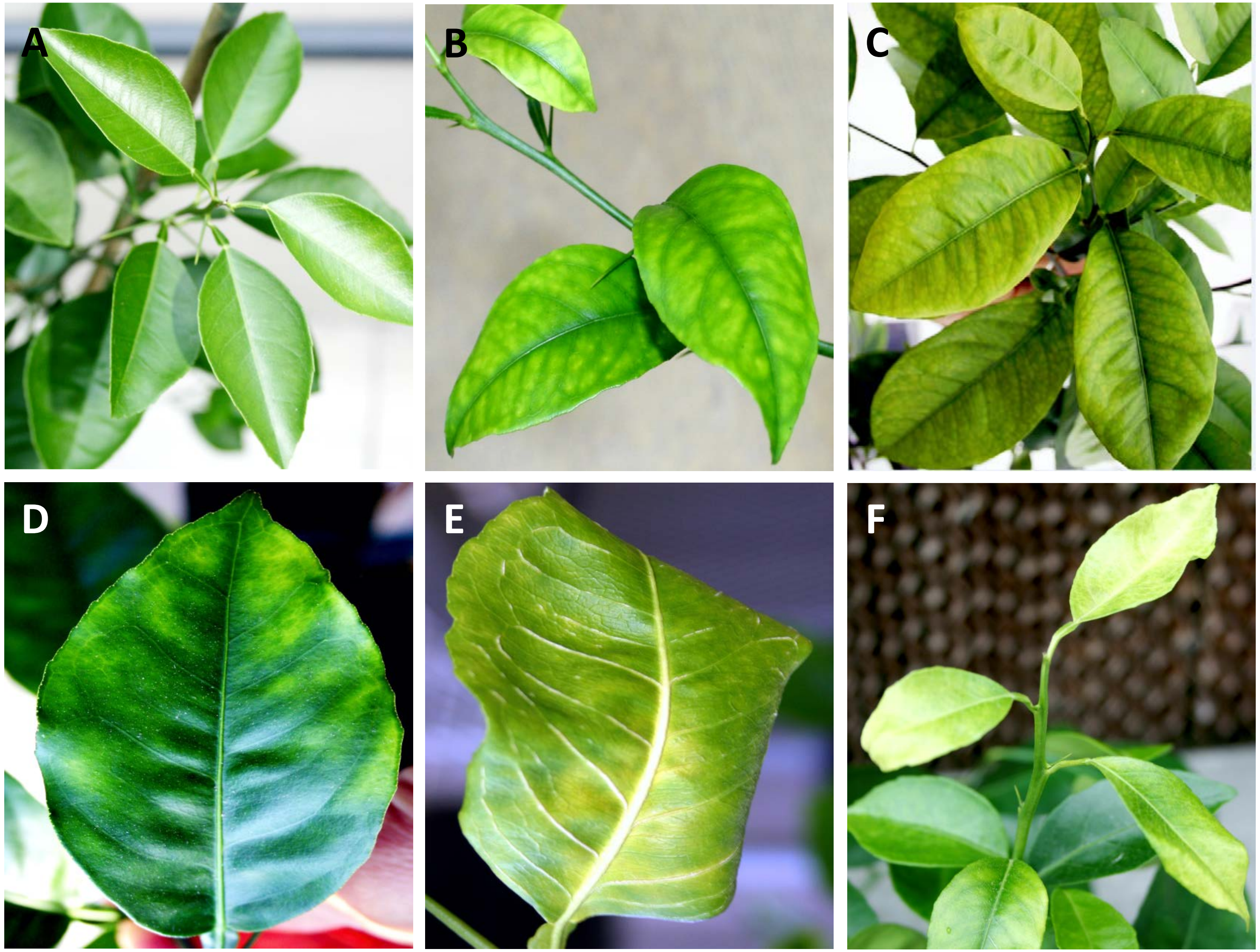

Figure S5. Typical leaf symptoms caused by CTV-B2, CTV-B6 or CaLas-B232 in single infection.
A, no symptoms caused by CTV-B2; **B** and **C**, chlorosis caused by CaLas-B232; **D**, **E** and **F**, chlorosis, leaf curing and vein corking symptoms caused by CTV-B6.
