# Supplementary material for: Transcriptional analysis of sweet orange trees co-infected with ‘Candidatus Liberibacter asiaticus’ and mild or severe strains of Citrus tristeza virus
Source: BMC Genomics. 2017 Oct 31;18:837. doi: 10.1186/s12864-017-4174-8 (PMC5664567; doi:10.1186/s12864-017-4174-8)
Supplement: Supplementary file 9 — Plant hormone pathways affected by co-infection with CTV-B2/CaLas-B232 and CTV-B6/CaLas-B232. Red boxes, down-regulated genes; Green boxes, up-regulated genes. (PDF 230 kb) [file 12864_2017_4174_MOESM9_ESM.pdf]

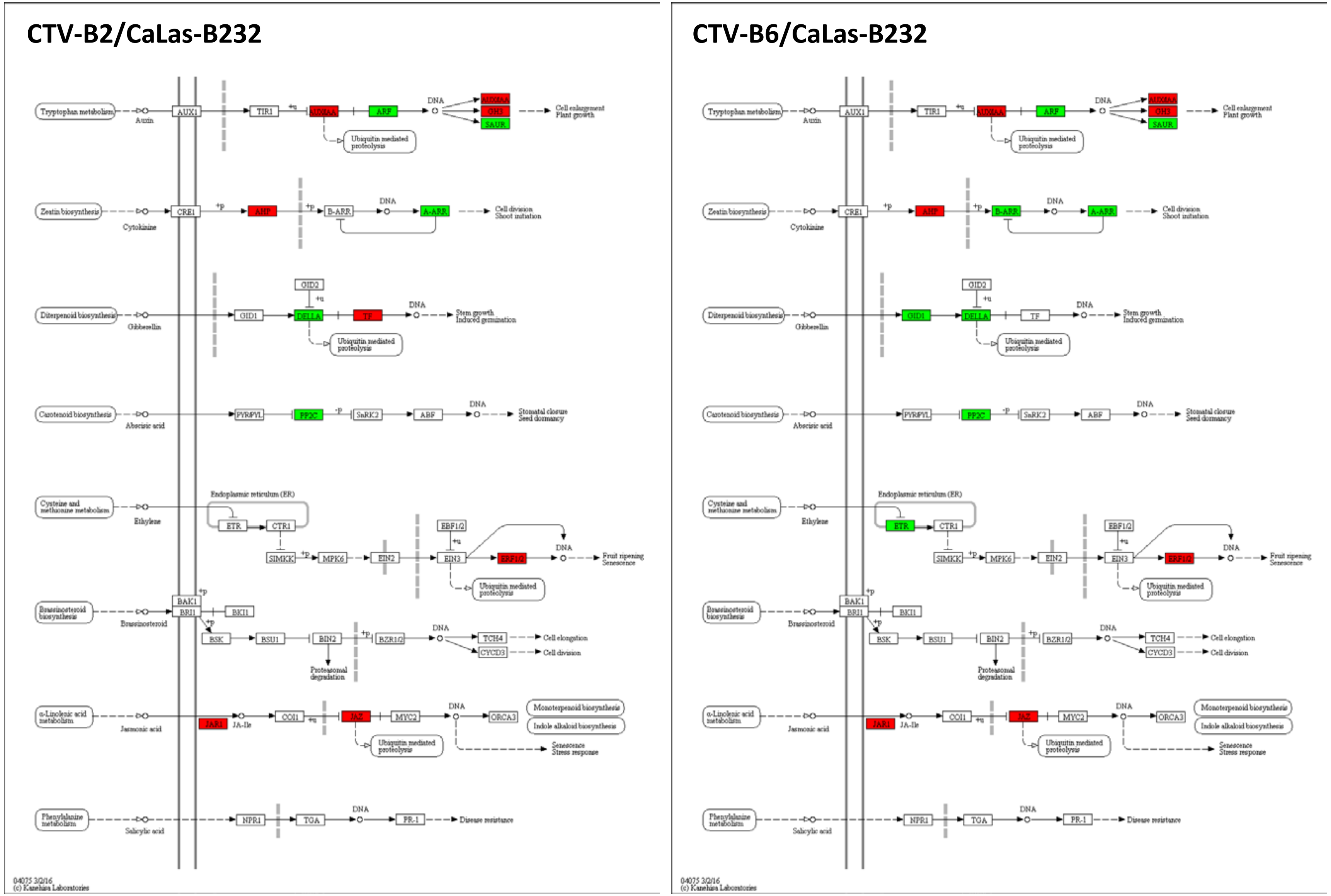

Figure S6. Plant hormone pathways affected by co-infection with CTV-B2/CaLas-B232 and CTV-B6/CaLas-B232. Red boxes, down-regulated genes; Green boxes, up-regulated genes.
