# Supplementary material for: Transcriptional analysis of sweet orange trees co-infected with ‘Candidatus Liberibacter asiaticus’ and mild or severe strains of Citrus tristeza virus
Source: BMC Genomics. 2017 Oct 31;18:837. doi: 10.1186/s12864-017-4174-8 (PMC5664567; doi:10.1186/s12864-017-4174-8)
Supplement: Supplementary file 10 — Expression of the actin gene estimated by RT-qPCR. HC, healthy control; B2/232, CTV-B2/CaLas-B232; B6/232, CTV-B6/CaLas-B232. Y-axis, Cq value. Bars with standard error of the mean expression level of the actin gene with three technical replicates for each biological replicate. (PDF 56 kb) [file 12864_2017_4174_MOESM10_ESM.pdf]

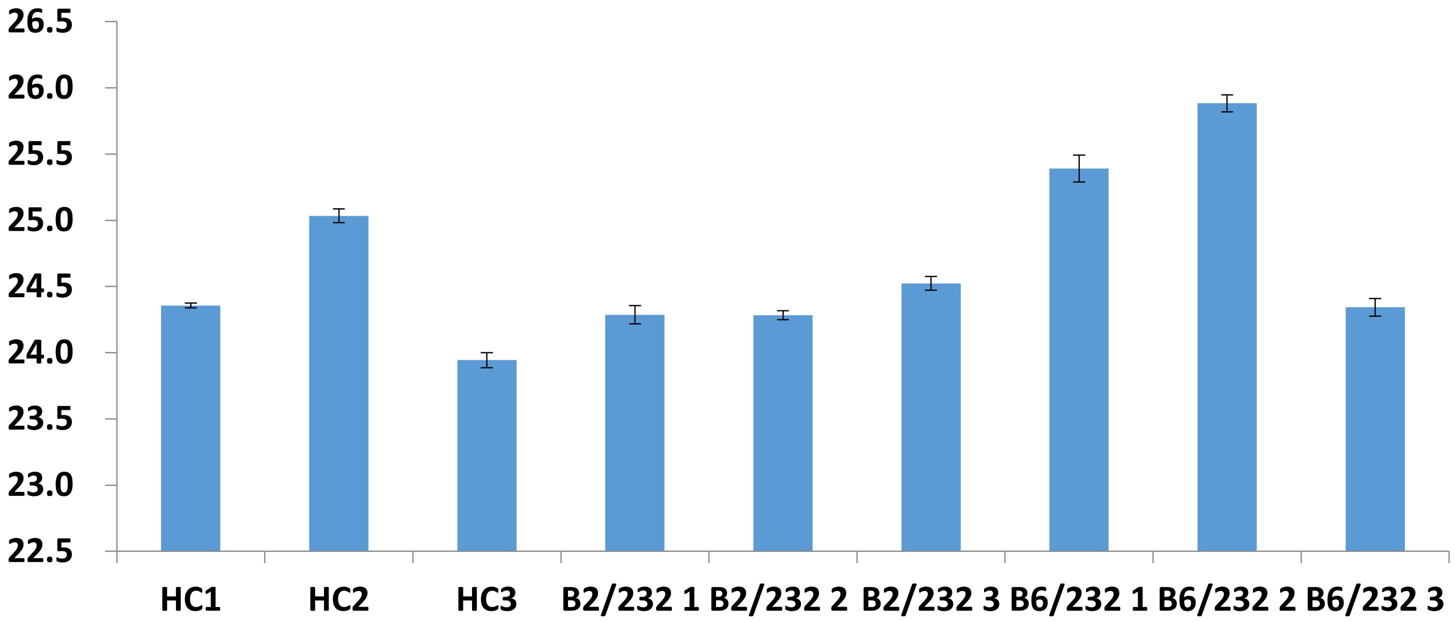

Figure S7. Expression of the actin gene estimated by RT-qPCR. **HC**, healthy control; **B2/232**, CTV-B2/CaLas-B232; **B6/232**, CTV-B6/CaLas-B232. Y-axis, Cq value. Bars with standard error of the mean expression level of the actin gene with three technical replicates for each biological replicate.
